# Mathematical Modeling Identifies Optimum Palbociclib-fulvestrant Dose Administration Schedules for the Treatment of Patients with Estrogen Receptor–positive Breast Cancer

**DOI:** 10.1158/2767-9764.CRC-23-0257

**Published:** 2023-11-16

**Authors:** Yu-Chen Cheng, Shayna Stein, Agostina Nardone, Weihan Liu, Wen Ma, Gabriella Cohen, Cristina Guarducci, Thomas O. McDonald, Rinath Jeselsohn, Franziska Michor

**Affiliations:** 1 Department of Data Science, Dana-Farber Cancer Institute, Boston, Massachusetts.; 2 Department of Biostatistics, Harvard T.H. Chan School of Public Health, Boston, Massachusetts.; 3 Center for Cancer Evolution, Dana-Farber Cancer Institute, Boston, Massachusetts.; 4 Department of Medical Oncology, Dana-Farber Cancer Institute, Boston Massachusetts.; 5 Department of Stem Cell and Regenerative Biology, Harvard University, Cambridge, Massachusetts.; 6 Center for Functional Cancer Epigenetics, Dana-Farber Cancer Institute, Boston, Massachusetts.; 7 Breast Oncology Center, Dana-Farber Cancer Institute, Boston, Massachusetts.; 8 Broad Institute of Harvard and MIT, Cambridge, Massachusetts.; 9 Ludwig Center at Harvard, Boston, Massachusetts.

## Abstract

Cyclin-dependent kinases 4/6 (CDK4/6) inhibitors such as palbociclib are approved for the treatment of metastatic estrogen receptor–positive (ER^+^) breast cancer in combination with endocrine therapies and significantly improve outcomes in patients with this disease. However, given the large number of possible pairwise drug combinations and administration schedules, it remains unclear which clinical strategy would lead to best survival. Here, we developed a computational, cell cycle–explicit model to characterize the pharmacodynamic response to palbociclib-fulvestrant combination therapy. This pharmacodynamic model was parameterized, in a Bayesian statistical inference approach, using *in vitro* data from cells with wild-type estrogen receptor (WT-ER) and cells expressing the activating missense ER mutation, Y537S, which confers resistance to fulvestrant. We then incorporated pharmacokinetic models derived from clinical data into our computational modeling platform. To systematically compare dose administration schedules, we performed *in silico* clinical trials based on integrating our pharmacodynamic and pharmacokinetic models as well as considering clinical toxicity constraints. We found that continuous dosing of palbociclib is more effective for lowering overall tumor burden than the standard, pulsed-dose palbociclib treatment. Importantly, our mathematical modeling and statistical analysis platform provides a rational method for comparing treatment strategies in search of optimal combination dosing strategies of other cell-cycle inhibitors in ER^+^ breast cancer.

**Significance:**

We created a computational modeling platform to predict the effects of fulvestrant/palbocilib treatment on WT-ER and Y537S-mutant breast cancer cells, and found that continuous treatment schedules are more effective than the standard, pulsed-dose palbociclib treatment schedule.

## Introduction

The advent of cyclin-dependent kinases 4/6 (CDK4/6) inhibitors (CDK4/6i) has transformed the treatment landscape in metastatic estrogen receptor–positive (ER^+^) breast cancer, and CDK4/6i in combination with endocrine treatment is now widely used as first- and second-line treatment in metastatic ER^+^ breast cancer (1). Currently, there are three approved CDK4/6i that have shown similar improvements in progression-free survival (PFS) in the metastatic setting: palbociclib (2), ribociclib (3), and abemaciclib (4). These three drugs differ in their scheduling and side effect profiles; while palbociclib and ribociclib are administered for 21 days followed by a 7-day break to allow for the recovery of the white blood cell count, abemaciclib is given twice daily continuously.

The current work focused on palbociclib, which received accelerated FDA approval based on the results of the PALOMA-1/TRIO18 trial (5). The trial showed that the addition of palbociclib to the aromatase inhibitor letrozole doubled PFS compared with letrozole alone in advanced ER^+^ breast cancer. Subsequently, the PALOMA-2 (2) and PALOMA-3 (6) phase III clinical trials showed a significant increase in PFS with the addition of palbociclib to either letrozole or fulvestrant in first- or second-line treatment of advanced ER^+^ breast cancer, respectively. The most common side effect of palbociclib is neutropenia. Although neutropenia in the setting of palbociclib is usually transient and manageable, it can require dose reductions, treatment interruptions or treatment delays, which could diminish the benefit of this drug.

Furthermore, intrinsic resistance to palbociclib is observed in approximately 15% of patients with metastatic ER^+^ breast cancer and ultimately nearly all patients will develop resistance (6). Several mechanisms of resistance to CDK4/6 inhibitors have been identified that can be categorized as (i) alterations in the CDK4/6-cyclin D1-pRb axis, such as increased expression of CDK6 and Rb1 mutations; (ii) upstream feedback adaptive mechanisms including activation of the PI3K-AKT and RAS-MAPK signaling pathways; and (iii) bypass downstream mechanisms including upregulation of cyclin E1 and CDK2 (reviewed in refs. 7–9). In addition, initial results from the PADA-1 trial showed that patients without clearance of an *ESR1* ligand-binding domain (LBD)-activating mutation had double the odds of disease progression on palbociclib and an aromatase inhibitor (10, 11). This type of mutation confers constitutive activity, resistance to estrogen deprivation and relative resistance to fulvestrant (12). In addition, the Y537S *ESR1-*activating mutation was found to be acquired after the development of resistance to fulvestrant in combination with palbociclib (13). These findings support the notion that sensitivity to endocrine treatment and the synergy between endocrine treatment and palbociclib are paramount to the benefit from this therapeutic combination. These clinical limitations raise the question of whether different treatment scheduling can improve tolerability and overcome intrinsic resistance or delay the development of acquired resistance to endocrine treatments in combination with palbociclib.

The question of how to schedule palbociclib in combination with endocrine treatment most effectively remains unresolved. This question is of particular relevance, because previous studies have shown that altering therapy administration dosage and schedules can substantially improve treatment outcomes (14, 15). In ref. 14, it was shown that an alternative schedule with lower doses of palbociclib and endocrine therapy administered more frequently reduced drug toxicities and still maintained plasma drug concentrations above the threshold required for efficacy. A phase III trial (15) compared the effects of administering 250 and 500 mg fulvestrant on outcomes in postmenopausal women with ER^+^ breast cancer and found that the higher dose led to an increase in PFS without increasing toxicity. Because of ethical and feasibility concerns, the entire space of possible combination dose administration schedules cannot be tested in clinical trials. Mathematical modeling in conjunction with careful parameterization, however, can be used to explore the effects of other possible combination treatment schedules and to predict the most effective schedule for reducing long-term tumor burden. These predictions can then be validated in preclinical trials before being tested in the clinic. We here sought to investigate whether there are alternative palbociclib dosing strategies that are more effective compared with the current standard of care while adhering to clinical constraints of feasible treatment schedules.

**FIGURE 1:**
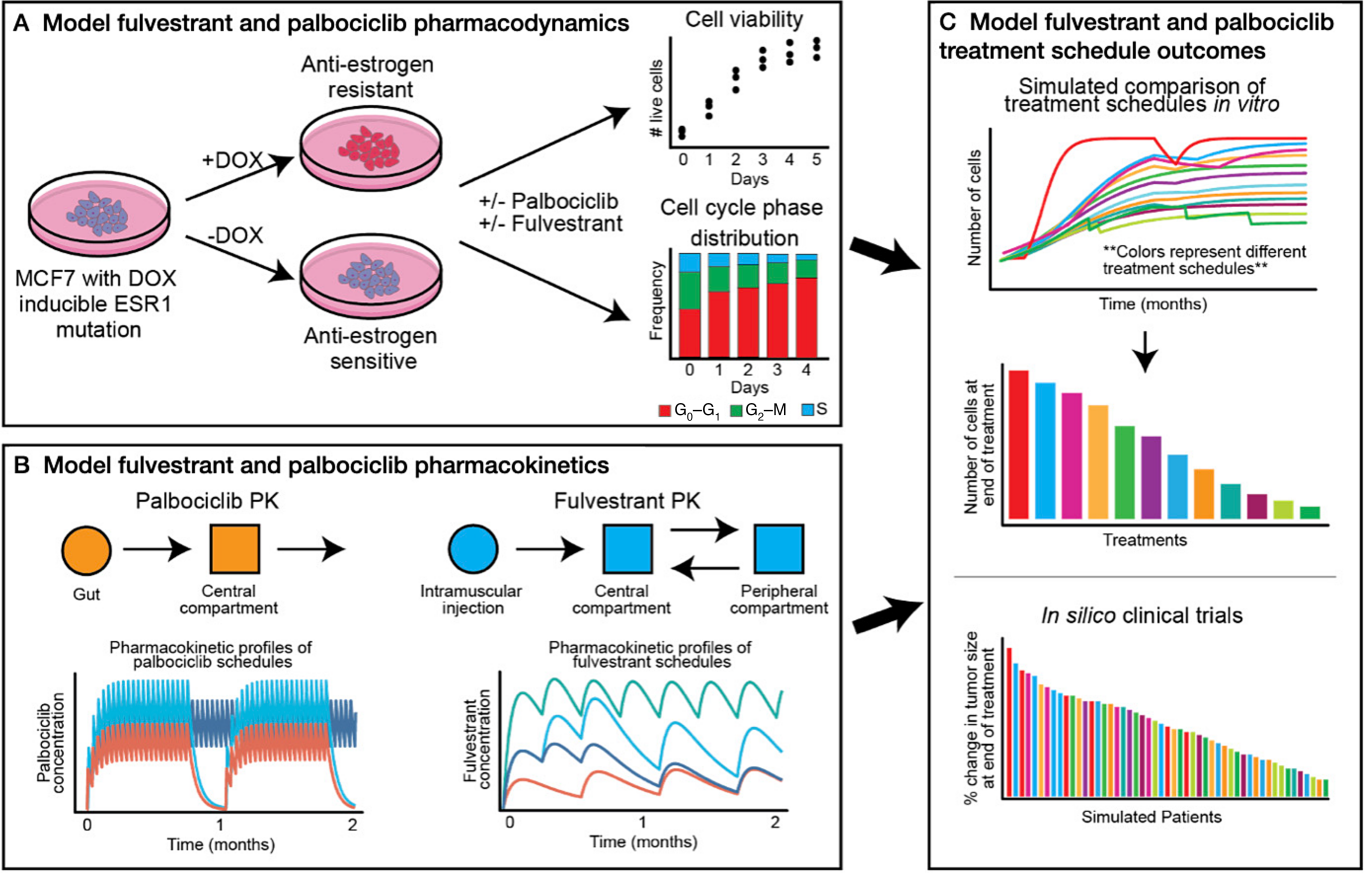
General framework of the combined experimental, mechanistic, and statistical modeling approach. **A,** Model of fulvestrant and palbociclib pharmacodynamics: we parameterized our mechanistic model using data from *in vitro* drug synergy and cell-cycle analysis assays in MCF7 −DOX and +DOX cells, respectively). **B,** Model of fulvestrant and palbociclib pharmacokinetics: we used the pharmacokinetic model derived from clinical data to describe the plasma concentration of fulvestrant and palbociclib in the blood stream. **C,** Model of fulvestrant and palbociclib treatment schedule outcomes: by the integration of our pharmacodynamic and pharmacokinetic modeling of fulvestrant and palbociclib treatment responses, we simulated *in silico* patient responses of different combination treatment schedules in patients with HER2^+^ breast cancer.

To this end, we interrogated different dosing strategies in ER^+^ breast cancer cells expressing either wild-type estrogen receptor (WT-ER) or doxycycline (DOX)-induced expression of the ER Y537S LBD-activating mutation, which is one of the most prevalent and potent *ESR1-*activating mutations (16). We adopted a multistage model of cell-cycle progression (17–20), combined with an effective drug dose model describing the extent of drug synergism (21) to model the response to fulvestrant plus palbociclib combination treatment for −DOX (WT-ER) cells and +DOX (expressing Y537S) cells, respectively. On the basis of data from *in vitro* drug synergy and cell-cycle assays, we then used Bayesian inference to estimate the model parameters. We sampled the model parameters from their posterior distributions to simulate the pharmacodynamics of palbociclib and fulvestrant, specifically their effect on G_1_ arrest, and further introduced a new drug-response metric defined in terms of the IC_50_ on the cell-cycle transition rate (TR_50_). Finally, we integrated the parameterized pharmacodynamic model with a pharmacokinetic model derived from clinical data to predict the optimal dosing schedules for reducing long-term tumor burden (Fig. 1).

## Materials and Methods

### Quick Guide to Equations and Assumptions

#### An Effective Drug Dose Model

We used a previously described drug dose–response model (21) to describe the interaction between fulvestrant and palbociclib. This model is an extension of the additive Bliss model (22, 23), which describes the combination drug response as the product of the single drug response curves and uses the effective doses that differ from the actual doses due to interactions with other drugs in the combination. In general, the effective concentration of each drug is given by

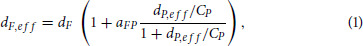

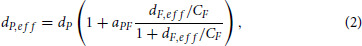


where *d_F_* and *d_P_* are the actual concentrations of fulvestrant and palbociclib, respectively; *C_F_* and *C_P_* are the doses of fulvestrant and palbociclib, respectively, causing 50% of the maximum drug response, where the latter is the theoretical maximum as the drug concentration approaches infinity and the drug response refers to the G_1_-to-S transition rate defined in Eqs. (3) and (4); and *a_FP_* and *a_PF_* are the interaction parameters between fulvestrant and palbociclib. Note that *a_FP_*, *a_PF_>* 0 when the two drugs are antagonistic, *a_FP_*, *a_PF_<* 0 when the two drugs are synergistic, and *a_FP_*, *a_PF_* = 0 when there is no interaction. To reduce the complexity of our model, we set the interaction parameter *a_PF_* in Eq. (2) to zero. This simplification was suggested in ref. 21, which showed that this model reduction by setting one of the two interaction parameters to zero gives very little loss of accuracy.

We then defined the G_1_-to-S transition rate, 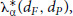, as 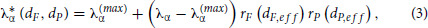


where *λ*^(^*^max^*^)^*α* is the G_1_-to-S transition rate as the amount of drug approaches infinity, and 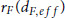 and 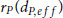 are the effective responses of fulvestrant and palbociclib, respectively, which are given by



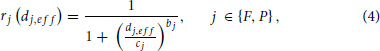



This choice is based on a previous study (24) demonstrating that the dose–response curves are well characterized by Hill curves.

#### A Cell Cycle–explicit Model

On the basis of the setup of the multistage cell-cycle model (Supplementary Appendix S1) and the G_1_-to-S transition rate defined in the effective drug dose model, the parameters and the phases of the chain of cell-cycle progression are specified as

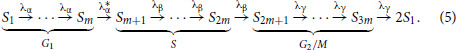


Given this chain of cell-cycle progression, our mathematical model is then specified as



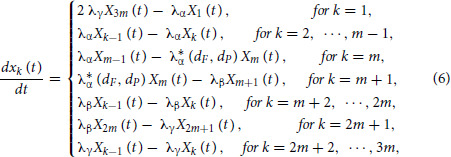



and according to the summation formulas (Supplementary Appendix S1)



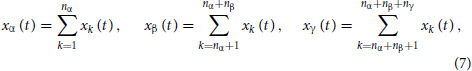



where 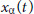 represents the number of cells in phase G_0_–G_1_ of the cell cycle, 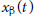 represents the number of cells in S phase, and 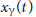 represents the number of cells in G_2_–M at time *t*, the system of differential Eq. (6) can be aggregated into three equations,



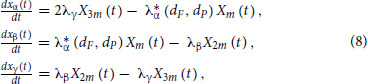



to model the cell dynamics in G_0_–G_1_, S, and G_2_–M phases, respectively. See Supplementary Fig. S1 for an illustration of our model [Eqs. (6)–(8)].

### Cell Culture

MCF7 cells (ATCC HTB-22) with DOX (catalog no. S631311)-inducible expression of the Y537S ER mutation were grown in DMEM supplemented with 10% FBS and 1% penicillin-streptomycin-glutamine, hereafter referred to as full media conditions. MCF7 cells were authenticated by short tandem repeat profiling (Bio-Synthesis) and regularly tested for *Mycoplasma* contamination using the MycoAlert Mycoplasma Detection Kit (Lonza) according to the manufacturer's instructions. The generation of the DOX-induced Y537S ER-mutant cells was previously described and these cells have been extensively characterized (16).

### Drug Synergy Analysis

MCF7 cells were grown in full media in the presence or absence of DOX for 3 days. On day −1, cells were plated in 96-well plates (Greiner 655090) in triplicates, with 2,000 cells/well for −DOX cells and 3,000 cells/well for +DOX cells. The numbers of cells plated per cell line was determined on the basis of the number of cells that allowed sufficient growth and avoided confluency on day 5 in vehicle conditions. On day 0, cells plated in the day 0 plate were counted. All other plates were treated on day 0 only. Treatment included vehicle and 2-fold serial dilutions for four doses of palbociclib (catalog no. S1579) at concentrations of 12.5, 25, 50, and 100 nmol/L, as well as fulvestrant (catalog no. S1191) at concentrations of 0.65, 1.3, 2.6, and 5.2 nmol/L, in a matrix format to include 25 different dose combinations. Cells were counted on days 1, 2, 3, 4, and 5. Viable cells were stained with Hoechst (catalog no. H3570) and counted using the Celigo image cytometer.

### Cell-cycle Analysis

MCF7 cells were grown in full media in the presence or absence of DOX for 3 days. On day 3, 500 cells/well were plated in 96-well plates (Greiner 655090) in triplicates for each condition. On day 1, cells were serum-starved for 24 hours for synchronization. The cell-cycle phase distribution was analyzed on day 0 in the day 0 plates, and all other plates were treated. Treatments were fulvestrant (F) at 1.25 nmol/L (F1), 2.5 nmol/L (F2), 5 nmol/L (F3), and 10 nmol/L (F4); palbociclib (P) at 12.5 nmol/L (P1), 25 nmol/L (P2), 50 nmol/L (P3), and 100 nmol/L (P4); and their combination (F1+P1, F2+P2, F3+P3, F4+P4). The cell-cycle phase distribution was analyzed on days 1, 2, 3, and 4. Cell-cycle analysis was done using the Click-iT EdU HCS Assay kit (Invitrogen, catalog no. C10351) as per manufacturer's instruction, with EdU staining for 4 hours at 37°C followed by scanning and analysis with the Celigo cytometer using the Target 1 (EdU) + Mask (DAPI) setting.

### Bayesian Estimation of Model Parameters

Data from *in vitro* drug synergy and cell-cycle assays were used to estimate all cell-cycle transition rates including the effective transition rate from G_1_ to S, 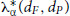 given by Eq. (3) under varying drug concentrations. Bayesian inference was used to estimate model parameters and the number of tumor cells on day 0 using the Stan programming language and CmdStan R package (25). Markov Chain Monte Carlo (MCMC) sampling was performed using the no-U-turn sampler (NUTS; ref. 26). Normal priors were used for all model parameters, while Half-Cauchy priors were used for the variance parameters as proposed in ref. 27. Details of choosing priors, generating posterior samples of the model parameters and the cell counts on day 0, and evaluating model inference are provided in Supplementary Appendix S2.

### Simulating *In Silico* Clinical Trials

We chose several treatment administration schedules (Table 1) as alternative continuous dosing schedules within current toxicity constraints based on evidence from recent phase II and III randomized trials (28, 29). To predict the outcomes of administering these treatment schedules, we simulated a cohort of 1,500 *in silico* patients according to the following two steps. In step one, we modeled the pharmacokinetics of *in silico* patients as informed by clinical trial data (30, 31): palbociclib pharmacokinetics were characterized by linear absorption kinetics, with time *t_max_* to reach the maximum plasma concentration *C_max_* and an exponential decay with half-life *T*_1_*_/_*_2_ (30), while fulvestrant pharmacokinetics were described using a two-compartment kinetic model (31). The pharmacokinetic parameters of each *in silico* patient were sampled from log-normal distributions with the mean values and the variances determined from the clinical trial data (30, 31). In step two, we sampled all parameters in the multistage cell-cycle model (Supplementary Table S1), including the day 0 values of tumor cell counts [Supplementary Appendix S2: Eqs. (4)–(7)], from their posterior distributions derived as described in the section *Bayesian Estimation of Model Parameters*. By generating this cohort of 1,500 *in silico* patients with the parameters sampled as described above, for each of the treatment schedules listed in Table 1, we simulated the drug concentrations over time derived from the pharmacokinetic model, and applied the simulated drug concentrations to Eqs. (6)–(8) of the cell-cycle model to predict the number of tumor cells for each patient. Treatment response dynamics of each *in silico* patient was simulated for a period of 100 days. The duration of this period was chosen such that the trend of the treatment response, in terms of the predicted number of tumor cells, was observable (Table 1) and lasted for more than one week (see Results section). Finally, the predicted number of tumor cells at day 100 was used to rank the proposed treatment schedules according to their performance (Table 1).

**TABLE 1:** *In silico* clinical trial schedules of palbociclib

Schedule name	Schedule description
Current standard schedule	3 weeks on, 1 week off of 125 mg daily
Daily 100 mg	Continuous daily dosing of 100 mg
Daily 75 mg	Continuous daily dosing of 75 mg
BID 50 mg	Continuous daily dosing of 50 mg administered twice per day (100 mg in total)
BID 50 mg in the morning, 25 mg at night	Continuous daily dosing of 50 mg in the morning, 25 mg at night (75 mg in total)

NOTE: All palbociclib schedules are in combination with the current standard administration schedule for fulvestrant (500 mg intramuscular on days 1, 15, and 29, and then monthly thereafter).

### Data Availability

All data are available from the authors upon request.

### Code Availability

All code used to process data and generate figures is available on a public Github repository at https://github.com/Michorlab/Optimal_Schedule_ER-positive.

## Results

### Characterization of the *In Vitro* Response to Combination Treatment

To characterize the *in vitro* response to fulvestrant-palbociclib combination treatment, we performed 5-day drug synergy experiments for four doses of palbociclib (12.5, 25, 50, and 100 nmol/L) and fulvestrant (0.65, 1.3, 2.6, and 5.2 nmol/L) in a matrix format to include 25 different dose combinations (Supplementary Fig. S2) and 4-day cell-cycle experiments for fulvestrant at 1.25 nmol/L (F1), 2.5 nmol/L (F2), 5 nmol/L (F3), and 10 nmol/L (F4); palbociclib at 12.5 nmol/L (P1), 25 nmol/L (P2), 50 nmol/L (P3), and 100 nmol/L (P4); and their combination (F1+P1, F2+P2, F3+P3, F4+P4; Supplementary Fig. S3) using MCF7 cells containing a Dox-inducible Y537S *ESR1* mutation, which confers relative resistance to fulvestrant (16). The choice of concentrations was made to guarantee that the range covers the IC_50_s for both −DOX and +DOX cells, defined by the cell count measured at day 5 in the drug synergy experiments (Table 2). We observed that cell growth was inhibited when the dose of either palbociclib or fulvestrant increased for −DOX cells (WT-ER; Supplementary Fig. S2A). On the other hand, we observed a marginal inhibitory effect of low-dose fulvestrant on the +DOX (Y537S-mutant) cells (Supplementary Fig. S2B). These observations are supported by the growth rates (GR) determined from the *in vitro* data (Supplementary Table S2). Furthermore, our data showed that the cells accumulating in G_0_–G_1_ led the growth to plateau in the overall population in a dose-dependent manner during treatment with palbociclib and fulvestrant (Supplementary Fig. S3). These observations were expected because fulvestrant and palbociclib prevent progression through the G_1_ to S checkpoint (32–34). In addition, at the lowest concentrations of the fulvestrant-palbociclib combination in the 4-day cell-cycle experiments (fulvestrant at 1.25 nmol/L in combination with palbociclib at a concentration of 12.5 nmol/L), we observed a lower level of G_0_–G_1_ accumulation with the induction of the Y537S mutation (Supplementary Fig. S3), consistent with the relative resistance to fulvestrant engendered by this mutation as observed in preclinical and clinical studies (11, 12, 35, 36). As the concentration of the combination increased to a higher level (fulvestrant 5 nmol/L + palbociclib 50 nmol/L), we observed that the level of G_0_–G_1_ accumulation of Y537S-mutant cells became similar to the level of WT-ER cells (Supplementary Fig. S3), suggesting that the higher concentration in the combination treatment may overcome resistance to fulvestrant.

**TABLE 2:** IC_50_s versus G_1_–S TR_50_ of palbociclib and fulvestrant

	Palbociclib (IC_50_)	Palbociclib (G_1_–S TR_50_)	Fulvestrant (IC_50_)	Fulvestrant (G_1_–S TR_50_)
−*DOX*	58 nmol/L	7.7 nmol/L	2 ∗ 10*^−^*^1^ nmol/L	1.47 ∗ 10*^−^*^5^ nmol/L
+*DOX*	41 nmol/L	18 nmol/L	5 nmol/L	1.49 nmol/L

NOTE: IC_50_s represent the concentrations at which the total cell growth counts are half the control at day 5 (Supplementary Fig. S2) and G_1_–S TR_50_s represent the concentrations at which the mean of transition rates from G_1_ to S are half the control.

### A Mathematical Model of Combination Treatment Response

On the basis of our current understanding of the mechanism of action of endocrine treatment and CDK4/6 inhibitors (1, 2, 32–34) and the data obtained from the cell culture experiments described above, we designed a mechanistic model that explicitly incorporates cell-cycle status to describe the response to fulvestrant plus palbociclib combination therapy. The model is based on a multistage process of cell-cycle progression, which consists of a set of linear ordinary differential equations (ODEs) [Eq. (6)] that models the number of cells in every phase of the cell cycle. By summing over the subphases of each cell-cycle phase, we obtained the three equations shown in Eq. (8) to describe cell dynamics in the G_0_–G_1_, S, G_2_–M phases of the cell cycle, respectively. We did not include carrying capacities in the model based on the observation in our control experiments (zero doses for both fulvestrant and palbociclib in Supplementary Fig. S2) showing that the cells treated with or without DOX displayed no obvious contact inhibition during cell growth within the 5-day duration of the experiment, an observation consistent with the evidence of loss of contact inhibition in cancer cells (37, 38). To justify this assumption, we compared the results of our model with those of a logistic growth model (39) to validate that the effects of carrying capacities are negligible for the parameter regimes estimated on the basis of our data (Supplementary Appendix S3).

As discussed in Materials and Methods, the model parameters of the transition rates are given by the chain of cell-cycle progression Eq. (5). The transition rates defined in the chain, Eq. (5), *λ_α_*, *λ_β_*, and *λ_γ_* are held constant and 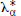 are modeled as a function of the drug concentrations of fulvestrant and palbociclib since the rate 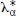 for the transition from G_1_ to S is set to be the only parameter affected by treatment, an assumption that is supported by several biological studies (32–34). For all model parameters, *λ_α_*, *λ_β_*, *λ_γ_*, and the set of parameters in function 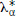 given by Eq. (3), we implemented a NUTS MCMC algorithm to infer the parameters that best fit cell growth and treatment response dynamics. The model was estimated using both *in vitro* drug synergy datasets from the 5-day drug synergy experiments (Supplementary Fig. S2) and the *in vitro* cell-cycle data from 4-day cell-cycle phase experiments (Supplementary Fig. S3).

Different cell lines may have a different number of subphases, *m*, in the multistage model (18–20). Therefore, we compared models with different values of *m* using the leave-one-out information criterion (LOOIC), using the **loo compare** function in **R** (40), to infer the most probable number of subphases of each phase for −DOX and +DOX cells, respectively. For model selection of the values of *m*, we chose integers from *m* = 1 to 10 as well as *m* = 20; the latter value was utilized to validate that 1 to 10 is a proper range. Among this set of values for *m*, we obtained a global maximum value for the model comparison; this value was not on the boundaries (1 and 10), and the value *m* = 20 led to significantly worse results as evaluated by LOOIC as compared with *m* = 10 (Supplementary Fig. S4). Using this approach, we estimated that the best value of *m* for −DOX cells was *m* = 8 and for +DOX cells *m* = 2 (Supplementary Fig. S4), thus suggesting that the Dox-inducible *ESR1* mutation may lead to a change in the number of the sequence of memoryless steps of the underlying biochemical reactions of cell-cycle progression. The correspondence between the memoryless steps and the number of subphases has been investigated in refs. 17–20. These studies showed that the cell-cycle progression defined by the G_0_–G_1_, S, and G_2_–M phases is not memoryless. However, by subdividing each phase into *m* rate-limiting steps (with the integer *m* varying among cell lines), the cell-cycle progression modeled by the progression on the subphases becomes a Markov process, in which each step is memoryless. For a large number of cells, the mean number of cells at each subphase follows the system of 3*m*-dimensional ODEs [Eq. (6)]. Therefore, our result of the difference in *m* between −DOX and +DOX cells implies that the *ESR1* mutation may lead to an underlying change in the rate-limiting steps of the cell cycle.

**FIGURE 2:**
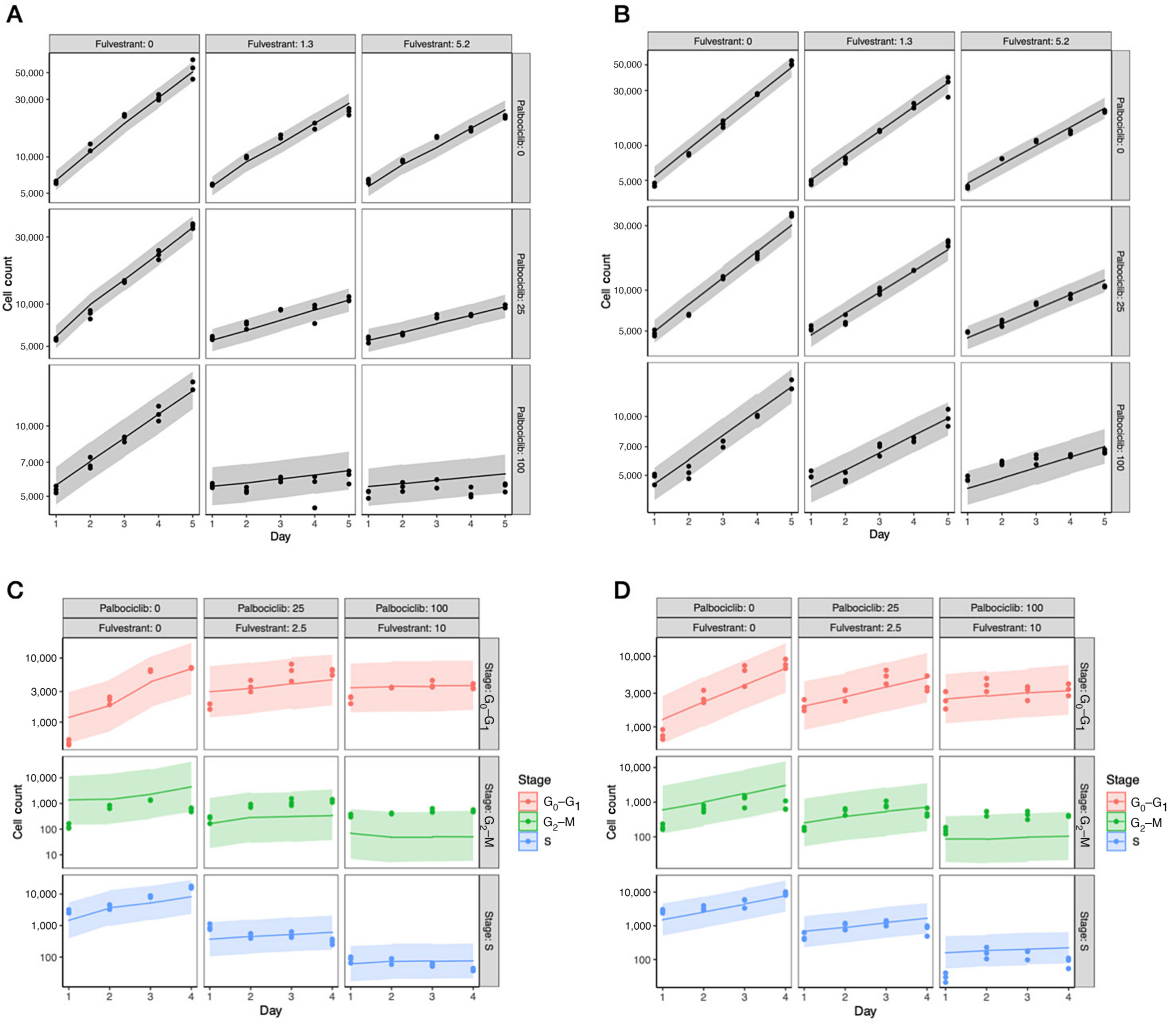
**A, B,** Posterior prediction of the total cell count. Each panel shows the posterior predicted total number of live cells over 5 days for a specific combination of palbociclib and fulvestrant for −DOX (A) and +DOX (B) cells. The line represents the median posterior predicted live cell count value over 5 days and the gray shaded area corresponds to the 95% credible interval of the posterior predictive values. The datapoints represent the observed cell counts from the drug synergy experiments used to train the model. The concentration of palbociclib increases across the columns and is denoted at the top of each column; the concentration of fulvestrant increases down the rows and is denoted to the right of each row. The unit of drug concentrations is nmol/L. **C, D,** The posterior predictive number of cells in each phase of the cell cycle for −DOX (C) and +DOX (D) cells treated with fulvestrant and palbociclib in combination. Each panel corresponds to a specific dose of fulvestrant and palbociclib. The line represents the median posterior predictive value and the shaded area corresponds to the 95% credible interval of the posterior predictive values. The datapoints represent the observed cell phase counts from the cell-cycle analysis experiments used to train the model. The concentrations of fulvestrant and palbociclib increase across the columns and are denoted at the top of each column. Red, blue, and green represent the G_0_–G_1_, S, and G_2_–M phases, respectively. See Supplementary Fig. S5 and S6 for more sets of drug concentrations.

We then used the posterior parameter values to generate posterior predictive samples of the cell count over 5 days of combination treatment. We found that the model accurately predicted the total cell count for both −DOX and +DOX cells with small 95% credible intervals of the posterior predictions, spanning 1*/*2 to 1*/*3 orders of magnitude of the medians (Fig. 2A and B for a representative subset and Supplementary Fig. S5 for details). In contrast, the 95% credible intervals of the posterior predictions of the cell-cycle phase analysis were large, often spanning 2 or 3 orders of magnitude of the medians, suggesting that the cell-cycle phase estimates had a larger degree of variability (Fig. 2C and D for a representative subset and Supplementary Fig. S6 for details). This uncertainty in the cell-cycle data likely arises due to the EdU assay's limitation to definitively differentiate between cells that are in the S versus G_2_–M phases of the cell cycle. As a result, about 10% of cells were between phases and were not assigned to any phase. Two approaches to address this limitation, one relating to the experimental design and the other based on modeling, are provided in the discussion section.

### Pharmacodynamics of Palbociclib and Fulvestrant

The IC_50_ is a common metric to quantify drug sensitivity and resistance (41). The standard approach to determining this conventional metric is based on cell counts obtained at the end of the experiment. However, during the entire time-course assays, cells may encounter various numbers of divisions due to variations in the control variables of the experiment, independently of the drug effects, and therefore IC_50_ might provide an incomplete picture of the drug response. To overcome this issue, other metrics such as the normalized growth rate (GR) inhibition (42) have been suggested. Here, in addition to determining IC_50_ and GR_50_, we were able to additionally infer the transition rates of the cell cycle as a function of the concentrations of palbociclib and fulvestrant. Estimating these rates based on the *in vitro* cell-cycle data enabled us to estimate the pharmacodynamics of palbociclib/fulvestrant; of particular importance was the drugs’ effect on controlling the transition from G_1_ to S. On the basis of recent studies (32–34), the drug inhibition of the G_1_–S transition leads to a reduction in the cell number and the GR. Therefore, for drugs such as palbociclib and fulvestrant that control the transition from G_1_ to S, the metric of a 50% reduction of the G_1_–S transition rate should be considered as a more direct measure of the drug effect than either IC_50_ (measuring cell number) or GR_50_ (measuring cell GR). To distinguish the 50% G_1_–S transition rate from the IC_50_ and GR_50_ metrics, we defined the term “G_1_–S TR_50_”, where TR represents the transition rate.

**FIGURE 3:**
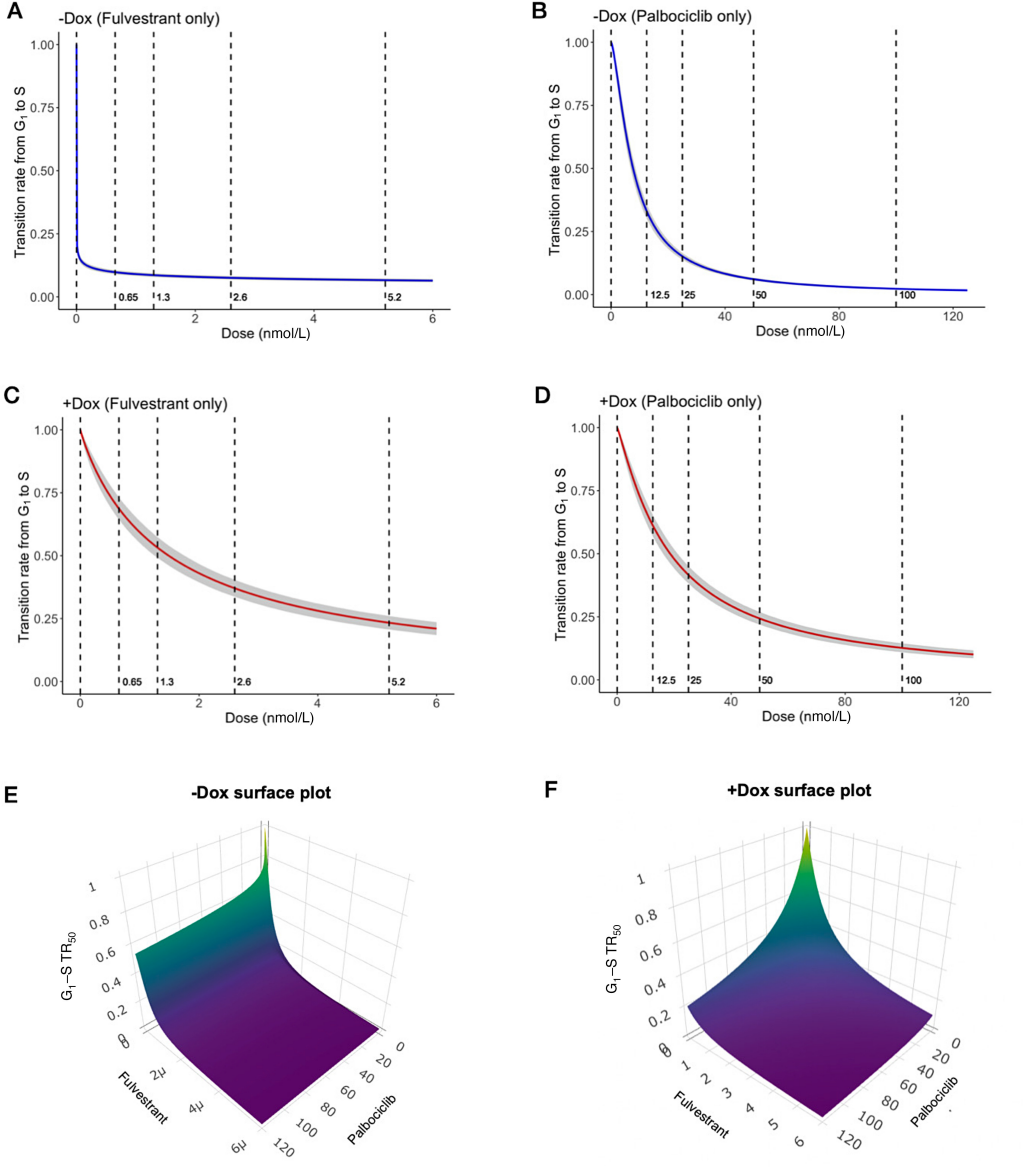
Pharmacodynamics of palbociclib and fulvestrant. **A**–**D,** In each panel, the *y*-axis represents the ratio of the G_1_-to-S transition rate treated by palbociclib/fulvestrant to the G_1_-to-S transition rate in control (zero drug concentration) and the *x*-axis represents drug concentrations in the unit of nmol/L. The gray shaded area corresponds to the 95% credible interval of the posterior predictive values. **A,** Fulvestrant only in −DOX cells. **B,** Palbociclib only in −DOX cells. **C,** Fulvestrant only in +DOX cells. **D,** Palbociclib only in +DOX cells. E and F are the surface plots for G_1_–S TR_50_ (*z*-axis) with respect to the combinations of palbociclib (*x*-axis) and fulvestrant (*y*-axis). **E,** For −DOX cells: because the response to fulvestrant is extremely sensitive, the unit is rescaled to 1*e^−^*^6^ nmol/L; palbocilib is in the unit of nmol/L. **F,** For +DOX cells: the units of palbociclib and fulvestrant are both in nmol/L.

We then investigated the pharmacodynamics of palbociclib/fulvestrant for −DOX/+DOX cells and simulated cell growth with respect to different combination treatment strategies. First, we observed that the Hill functions, which represent the ratio of the G_1_-to-S transition rates of cells during treatment and the transition rate of cells in the control condition, change much more significantly between −DOX and +DOX cells in response to fulvestrant than in response to palbociclib (Fig. 3A–D). This result is consistent with the evidence that the *ESR1* mutation, in our experimental system induced by DOX, causes endocrine treatment–resistant breast cancer (16). Second, given data from *in vitro* drug synergy and cell-cycle assays and the Bayesian estimations of model parameters (Materials and Methods), we estimated the G_1_–S TR_50_s and the interaction parameter of palbociclib and fulvestrant (*a_FP_* defined in the effect drug dose model in the section of equations and assumptions). By drawing the samples of the posterior distributions derived from the Bayesian estimations, we plotted the densities of G_1_–S TR_50_s (Supplementary Fig. S7A–S7D) and the interaction parameter of palbociclib and fulvestrant (Supplementary Fig. S7E and S7F) for −DOX and +DOX cells, respectively. Negative values for the interaction parameter indicate synergism between palbociclib and fulvestrant while positive values show antagonism. From the posterior distributions, we inferred that the mean values of the interaction parameters were *−*0.113 and *−*0.1 for −DOX and +DOX cells, respectively, and over 87% of the samples were negative, suggesting that these two drugs are predominantly synergistic in probability based on the Bayesian estimations. Third, we provided surface (Fig. 3E and F) and contour plots (Supplementary Fig. S8) to describe the response surfaces of palbociclib and fulvestrant combinations. We found that −DOX cells are extremely sensitive to fulvestrant compared with palbociclib, as demonstrated by the asymmetric surface plot, but +DOX cells have the same level of response to palbociclib and fulvestrant, as indicated by the symmetric surface plot. Finally, we compared the means of G_1_–S TR_50_s and the IC_50_s determined by the concentrations at which the cell count is half that of the control condition at day 5 (Table 2): by computing the ratio of the value of fulvestrant to the value of palbociclib in −DOX cells (a smaller ratio corresponds to a lower dose of fulvestrant to achieve the same effect of a unit dose of palbocilib), we found that the G_1_–S TR_50_ response metric is more sensitive compared with the conventional metric IC_50_; the G_1_–S TR_50_ has a ratio of 1.47 *×* 10*^−^*^5^*/*7.7 and the IC_50_ has a ratio 2 *×* 10*^−^*^1^*/*58. Moreover, by computing the ratio of the value of fulvestrant in +DOX cells to the value of fulvestrant in −DOX cells, we found that the *ESR1* mutation leads to about 10^4^ to 10^5^ in this ratio defined by G_1_–S TR_50_, but only 10^0^ to 10^1^ in this ratio defined by IC_50_, that is, the G_1_–S TR_50_ response metric provides a more significant change before and after acquiring the resistance to fulvestrant.

### Determining an Optimal Treatment Schedule for Clinical Testing

**FIGURE 4:**
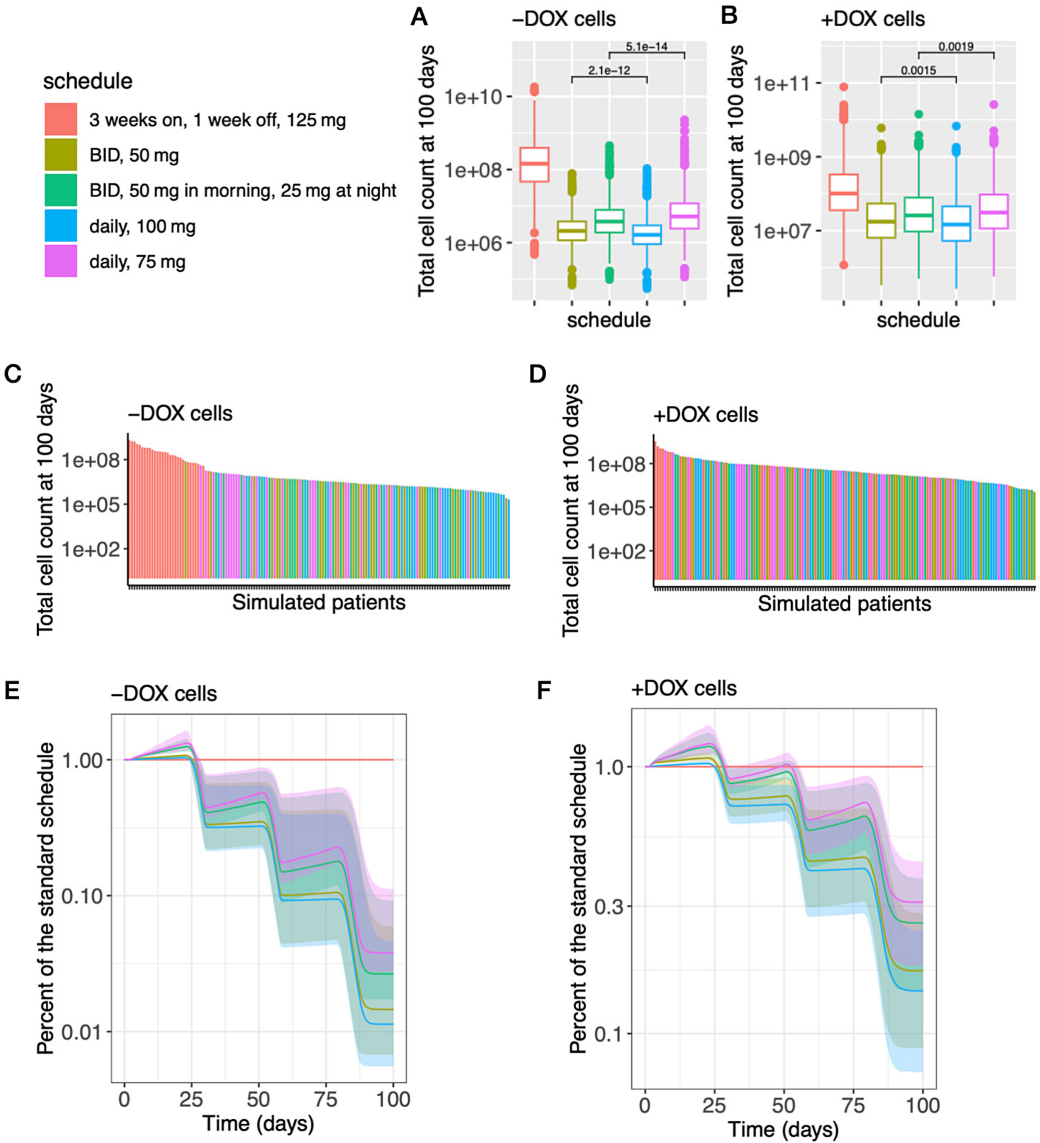
*In silico* trial predictions of multiple palbociclib treatment administration schedules in combination with fulvestrant. For −DOX cells (**A**) and for +DOX cells (**B**), The panels show the box plots for the number of cells at day 100. The *p*-values were computed using the Wilcoxon test. In each panel, we only show the largest two *p*-values. The *p*-values not shown in the panels, for the rest of pairs of treatment schedules, are all smaller than 1*e* − 15 in A and 1*e* − 13 in B. For −DOX cells (**C**) and for +DOX cells (**D**), The panels show the waterfall charts for 150 random samples from the group of 1,500 simulations in descending order of cell number at day 100. For −DOX cells (**E**) and for +DOX cells (**F**), The panels show the predictions of *in silico* cell growth trajectories by taking the ratio of each schedule to the standard schedule (daily, 125 mg, 3 weeks on, 1 week off). The shaded area corresponds to the 95% credible interval of the posterior predictive values.

We then set out to determine optimum treatment schedules for clinical use. To this end, we incorporated pharmacokinetic models into our modeling platform to predict the dynamics of drug response in 1,500 *in silico* patients (see Materials and Methods). We tested the schedules listed in Table 1; these schedules were chosen because they represent alternative continuous dosing schedules that are within current toxicity constraints suggested by the pooled analysis of the frequency of hematologic adverse events in the PALOMA clinical trials (29). For each of the 1,500 patients, we simulated 100-day treatment schedules and compared the predicted number of tumor cells of each schedule with the current standard palbociclib schedule (Fig. 4). The current standard palbociclib schedule is 125 mg per day for 3 weeks, followed by a 1-week treatment holiday, and all palbociclib schedules were in combination with the current standard-of-care fulvestrant schedule (500 mg monthly, with a 500 mg loading dose at day 14). Our *in silico* simulations involve administering palbociclib in conjunction with the current standard schedule for fulvestrant, which includes intramuscular injections of 500 mg on days 1, 15, and 29, followed by monthly injections thereafter. Our pharmacokinetic model, based on previously published data (31), demonstrates that there is an initial increase in plasma concentration of fulvestrant following intramuscular injection, which is subsequently followed by a decrease until the next injection (Supplementary Fig. S9A). This variability in plasma concentration is in agreement with the findings of two phase III trials (31), indicating that the concentration is not constant over time.

Our *in silico* clinical trial results suggest that continuous dosing of palbociclib is more effective in reducing the predicted overall tumor cell count than the standard, pulsed-dose palbociclib (Fig. 4A and B: *p* < 1*e* − 13 for each continuous dosing to the standard dosing at day 100, Wilcoxon test), the waterfall charts in descending order of the cell numbers at day 100 (Fig. 4C and D), and the trend of cell growth trajectories (Fig. 4E: growth pattern of −DOX cells after the first cycle, 28 days, of the treatment schedules; Fig. 4F: growth pattern of +DOX cells after the second cycle, 56 days, of the treatment schedules). On the basis of the clinical trial (30), our pharmacokinetic model indicated a decline in the plasma concentration of palbociclib when treatment was stopped every 3 weeks (Supplementary Fig. S9B). Our *in silico* predictions, combined with the pharmacokinetic model results, suggested that this decrease in palbociclib concentration upon treatment discontinuation was associated with a rebound to exponential growth. In addition, we found that one dose of palbociclib 100 mg per day is predicted to be more effective in reducing the overall tumor cell count than two doses of palbociclib 50 mg per day (Fig. 4A–D: the cell count at day 100 and Fig. 4E and F: the patterns of growth trajectories). Corresponding to this finding, we observed that the peak plasma concentration of palbociclib is larger when 100 mg of palbociclib is administered once per day in comparison with when 50 mg of palbociclib is administered twice a day (Supplementary Fig. S9B). In addition, because the lower bounds of the plasma concentrations of palbociclib given by the 100 mg daily schedule and the BID 50 mg (50 mg twice a day) schedule are similar, the higher peak plasma concentration of palbociclib leads to a higher mean plasma concentration (the ratio of 100 mg daily to BID 50 mg is 1.12). Interestingly, we found that a daily dose of 75 mg is predicted to be less effective in reducing the predicted number of tumor cells than splitting the dose into 50 mg in the morning and 25 mg at night (Fig. 4A–D: the cell count at day 100 and Fig. 4E and F: the patterns of growth trajectories). In distinction to the case of 100 mg daily versus 50 mg twice a day, we did not observe a difference of the peak plasma concentrations of palbociclib between the daily dose of 75 mg and splitting the dose into 50 mg in the morning and 25 mg at night (Supplementary Fig. S9); on the other hand, we observed that the lower bound of the plasma concentration of palbociclib was higher when splitting the dose into 50 mg in the morning and 25 mg at night (BID 50 mg/25 mg) in comparison with 75 mg of palbociclib administered once per day, that is, the plasma concentration of palbociclib does not drop as much in the BID 50 mg/25 mg schedule compared with the 75 mg schedule. Taken together, the mean plasma concentration of palbociclib is higher in the BID 50 mg/25 mg schedule (the ratio of BID 50 mg/25 mg to 75 mg daily is 1.07). On the basis of the results of daily dose versus BID dose in both 100 mg and 75 mg per day schedules, we found that maintaining a higher steady state mean plasma concentration, confined by the toxicity constraint, is associated with a higher efficacy in reducing the number of tumor cells.

We further investigated the proposed schedules (Table 1), particularly the 100 mg daily and BID 50 mg/25 mg per day schedules, to identify the optimal approach when considering adverse events. A clinical trial (43) showed that continuous dosing of palbociclib 100 mg led to a large rate of grade 3 and 4 neutropenia requiring dose reductions or dose delays in 70% of patients, and another clinical trial (29) found that continuous daily dosing of 100 mg of palbociclib led to a high rate of adverse events such that 33.8% of the patients required a palbociclib dose reduction from 100 to 75 mg. It is likely that the 75 mg per day schedules of palbociclib will have a lower rate of adverse events. We thus compared the treatment efficiency of the daily 100 mg and the BID 50 mg/25 mg schedules, and found that the daily 100 mg schedule is more effective in reducing the predicted tumor cell count at day 100 for either −DOX or +DOX of cells (Fig. 4A and B: *p* < 1*e* − 13, Wilcoxon test). In particular for +DOX cells, almost all (>95%) of the *in silico* patients with daily 100 mg dosing had less than one fourth of the cells at 100 day relative to the standard schedule (Fig. 4F). In contrast, under half (44.2%) of the *in silico* patients with BID 50 mg/25 mg achieved this fraction (Fig. 4F). However, the BID 50 mg/25 mg schedule is still more effective than the standard treatment schedule for either −DOX or +DOX cells (Fig. 4A and B: *p* < 1*e* − 13, Wilcoxon test). Taken together, our results suggest the following schedules to be best: (i) the BID 50 mg/25 mg of palbociclib for patients with decreased tolerability or (ii) daily 100 mg of palbociclib for patients with a large percentage of cells harboring a Y537S ER mutation (+DOX cells) and who experience manageable adverse events.

**FIGURE 5:**
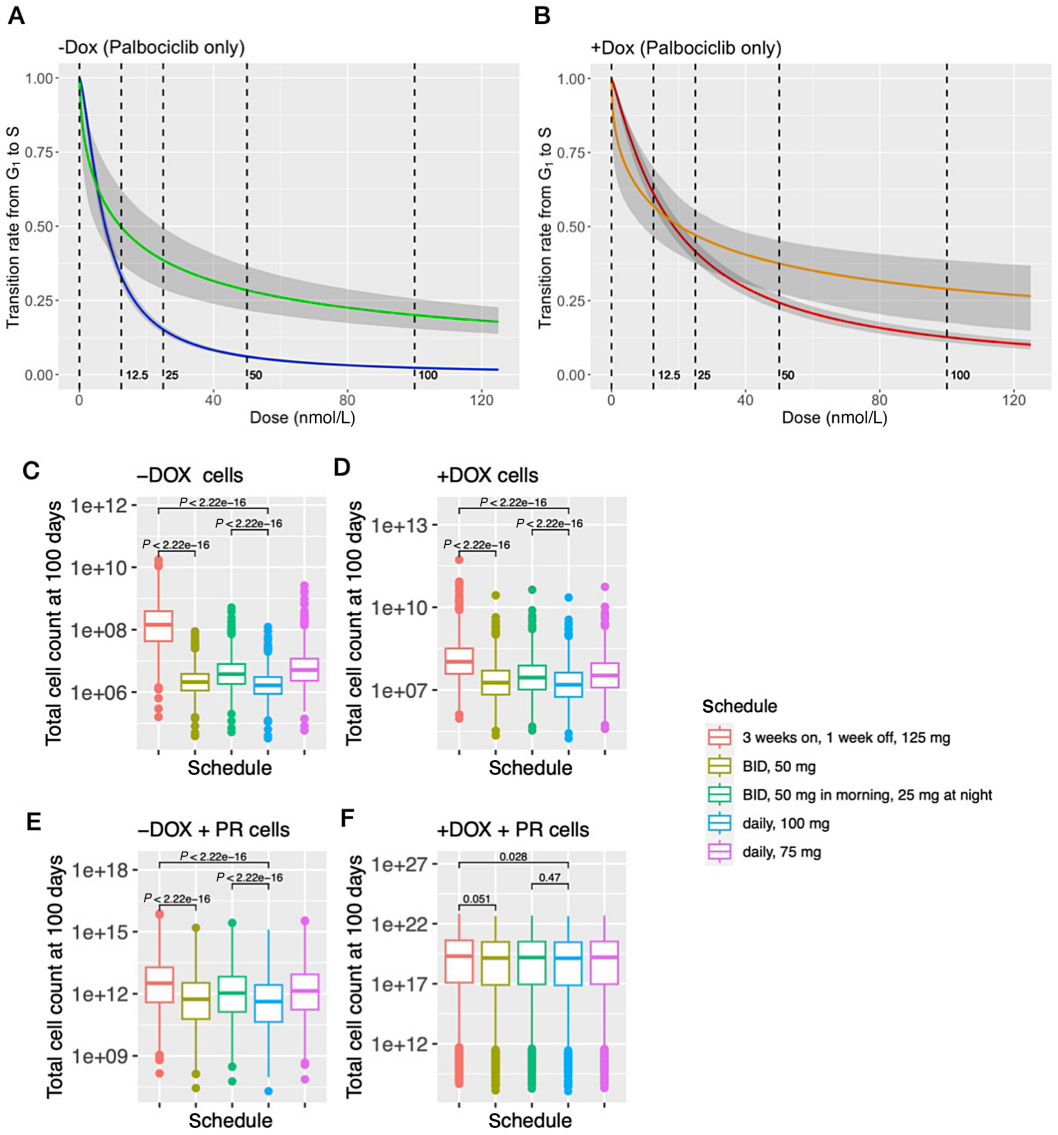
Prediction of drug responses in palbociclib-resistant cells. For −DOX cells (**A**) and for +DOX cells (**B**) are the estimated drug response curves of the effect of palbociclib on G_1_–S transition rate. The blue (−DOX) and red (+DOX) curves are given by the estimated model parameter of the palbociclib-sensitive MCF7 cell line. The green (−DOX) and orange (+DOX) curves are given by the estimated model parameter of the palbociclib-resistant MCF7 cell line. The gray shaded area corresponds to the 95% credible interval of the posterior predictive values. **C**–**F** show the box plots for the number of cells at day 100 given by the *in silico* trial predictions of multiple palbociclib treatment administration schedules in combination with fulvestrant. The *p*-values were computed using the Wilcoxon test. C and D are −DOX/+DOX cells of the palbociclib-sensitive MCF7 cell line. E and F are −DOX/+DOX cells of the palbociclib-resistant MCF7 cell line (+PR).

We then sought to investigate situations in which −DOX/+DOX cells acquire different levels of palbociclib resistance since clinical and preclinical trials have suggested that several specific molecular characteristics (e.g., RB loss and intrinsic subtypes) may lead to palbociclib resistance (13, 44, 45) and resistance to CDK4/6 inhibitors is a significant clinical challenge (7–9). We applied our mathematical model to the *in vitro* data of palbociclib-resistant MCF7 cells whose resistance was confirmed by a growth study (Supplementary Fig. S10). The drug response curves estimated from the data revealed that those cells are resistant to the effect of palbociclib with regard to its regulation of the G_1_–S transition rate (Fig. 5A and B). We then combined the pharmacodynamic model of this palbociclib-resistant cell line with the palbociclib pharmacokinetic model to perform *in silico* clinical trials (Table 1). We found that when the cells acquire only one type of drug resistance (either to fulvestrant or to palbociclib), continuous 100 mg treatment schedules (both 100 daily and 50 mg twice a day) are still significantly more efficient than the standard pulsed schedule: the continuous treatment schedules led to less than 20% of the predicted number of tumor cells at the end of 100 days of simulation compared with the standard pulsed schedule (*p* < 2.22e −16; Fig. 5C–E; Supplementary Fig. S11). However, as the cells acquire both fulvestrant and palbociclib resistance, only the 100 mg daily treatment schedule is significantly better than the standard treatment schedule. The predicted number of tumor cells at the end of 100 days of simulation using 100 mg daily dosage is 65.15% of the number of tumor cells predicted from the standard treatment schedule (*p* = 0.028; Fig. 5F; Supplementary Fig. S11).

To further identify the key parameters that significantly influence the optimal treatment schedule, we used a grid search of model parameters to simulate scenarios in which cells develop various levels of palbociclib resistance. This approach was adopted not only to investigate the dynamics of the resistant MCF7 cell line, but also other cell lines with various characteristics such as different G_1_–S transition rates in the absence of treatment as well as different IC_50_s and slopes of the palbociclib response curves. We tested different weights for those parameters that were inferred from −DOX/+DOX MCF7 cells to determine the appropriate range and combination of the parameters for simulating palbociclib resistance. The selection of parameters was deemed reasonable by comparing the growth of these simulated cells with the growth study of the palbociclib-resistant MCF7 cell line as a specific example (Supplementary Fig. S12). We then compared the *in silico* trial response predictions of these simulated cell lines and found that for almost all cell lines, the continuous treatment schedules remained more effective than the standard pulsed schedule (Supplementary Fig. S13–S15). The only exception was a cell line with a large increase of the G_1_–S TR_50_s (defined as concentrations leading to a 50% reduction in the rate of the G_1_–S transition) in the palbociclib response curve: when the G_1_–S TR_50_ value was inferred from the original palbociclib-sensitive MCF7 cells, the predicted number of tumor cells at the end of the continuous 100 mg treatment schedules was approximately 15% of the predicted number from the standard pulsed treatment schedule. However, when the G_1_–S TR_50_ was increased to 10 times the inferred value, the predicted number of tumor cells increased to around 50% of the predicted number from the pulsed treatment schedule. Finally, when the G_1_–S TR_50_ was 100 times the inferred value, this percentage increased to approximately 95% (see Supplementary Fig. S14 and Supplementary Table S3). We thus identified the key parameter leading to the lesser difference between the continuous treatment schedules and the pulsed schedule as the G_1_–S TR_50_s.

To account for changes in tumor heterogeneity, we generalized our *in silico* clinical trials to simulate the growth of the tumor using different initial proportions of WT-ER and ER-mutant cells. We varied the initial condition so that the ER-mutant population represented 0%, 20%, 50%, 80%, and 100% of the population. This simulation of tumor heterogeneity was repeated for the different cell lines (Supplementary Fig. S12). In general, we found that as the proportion of ER-mutant cells in an individual tumor increases, the treatment response dynamics become closer to the case of 100% +DOX cells, and vice versa. Therefore, if the optimal treatment schedules are the same for both homogeneous −DOX and +DOX tumors, then tumor heterogeneity does not change the conclusion about the efficacy of different treatment schedules. For instance, in the palbociclib-sensitive MCF7 cell line, continuous treatment schedules are more effective than the pulsed schedule when tumors are homogeneous, and we obtained the same conclusion for heterogeneous tumors (Supplementary Fig. S16). However, if we have different conclusions of optimal schedules for homogeneous +DOX and −DOX tumors, then the optimal schedule of a heterogeneous tumor depends on its initial percentage of −DOX cells. For instance, in the palbociclib-resistant cell line, we found that, when the percentage of −DOX cells is small, the 100 mg continuous dose schedules are significantly more effective than the pulsed schedule, similar to the findings in a homogeneous −DOX tumor. As the percentage of +DOX cells increases, the difference between the continuous dose schedules and the pulsed schedule becomes less pronounced, similar to the findings of homogeneous +DOX tumors (Supplementary Fig. S16).

## Discussion

To study the optimal scheduling of palbociclib in combination with fulvestrant with the aim of improving patient outcomes with this treatment combination, we used a cell cycle–explicit model of ER^+^ breast cancer response to these drugs, parameterized using *in vitro* drug synergy and cell-cycle data. Our approach led to the findings of suggested schedules that administer (i) palbociclib 50 mg/25 mg twice a day to patients with decreased tolerability or (ii) palbociclib 100 mg daily to patients with a large percentage of cells harboring a Y537S ER mutation and who experience manageable adverse events. Importantly, our approach may also be applicable to the schedule optimization of combination treatments with other cell-cycle inhibitors, including ribociclib and abemaciclib, the other two CDK4/6i that are FDA approved and are in clinical practice, as well as novel cell-cycle inhibitors in clinical development such as CDK2, CDK4 (46), CDK2/4/6 (9), or CDK7 inhibitors (47).

The NeoPalAna clinical trial (48) was a neoadjuvant study in which 50 patients with early-stage ER^+^/HER2-negative disease received 4 weeks of treatment with anastrozole (endocrine therapy) alone followed by four cycles of anastrozole in combination with palbociclib. Ki67 suppression based on the rate of complete cell-cycle arrest was significantly higher after 2 weeks of palbociclib in combination of anastrozole versus 4 weeks of anastrozole alone. However, the Ki67 levels in the tumors obtained at the time of surgery from the first 29 patients who completed four cycles of the anastrozole plus palbociclib following a median washout of 29 days (range, 8–49 days) prior to surgery were significantly higher compared with the on treatment Ki67 levels after 2 weeks of the combination treatment. The subsequently enrolled patients (*N* = 8) received 10–12 days of palbociclib immediately prior to surgery. In the patients that were treated with palbocicilib up until surgery, the degree of Ki67 suppression at the time of surgery was comparable to day 15 of the combination treatment. In addition, serum thymidine kinase activity, a potential surrogate marker for CDK4/6 inhibition, was also significantly decreased after 2 weeks of palbociclib and anastrozole in the NeoPalAna trial, but after withdrawal of palbociclib at the time of surgery there was a significant increase in the level of serum thymidine kinase activity (49). In line with these clinical observations, in preclinical experiments with xenografts of MCF7 cells and an ER^+^ patient-derived xenograft (PDX) model, when treatment was withdrawn, the tumors grew back and reached tumor volumes that were approximately 2.3-fold larger (50). These preclinical findings align with our *in silico* modeling predictions of a 3-fold change, offering supportive partial validation for our modeling approach. Taken together, these results indicate that continuous therapy is required for cell-cycle inhibition and tumor suppression.

Early results of a clinical trial testing a continuous dose of palbociclib 100 mg versus the standard regimen of 125 mg given for 21 days followed by a 7-day break did not show improvements in PFS (NCT02630693; ref. 43). However, the continuous dosing of palbociclib 100 mg led to a high rate of grade 3 and 4 neutropenia requiring dose reductions or dose delays in 70% of patients and limits the evaluation of the benefit with continuous scheduling. The 75 mg dose continuously is likely to be better tolerated and our results provide a rationale for testing the 75 mg dose given daily in split doses of 50 mg in the morning and 25 mg in the evening.

Even though our *in silico* predictions of the optimal treatment schedule suggest that continuous therapy is more effective than pulsed-dose therapy, these conclusions are only partially validated by existing preclinical and clinical trials. The general applicability of our findings, particularly the effects across various continuous treatment schedules, remains a limitation of our current work. To validate our full set of predictions, we propose the design of an ER^+^ PDX model, treated in line with our proposed treatment schedules.

Our model was parameterized using data solely from the MCF7 cell line, which is a caveat of this study. However, we have expanded the model's applicability by conducting a grid search over a range of parameters for drug resistance, confirming its relevance to different drug resistance mechanisms, such as resistance to both fulvestrant and palbociclib. In the future, we intend to apply our computational modeling platform to the *in vitro* cell-cycle data of other cell lines, such as the T47D line (16). This approach will enable us to obtain relevant parameters for the pharmacodynamic models. When merged with our current pharmacokinetic model and combined with the *in silico* prediction techniques from this research, we expect to be able to further optimize treatment schedules based on a broader array of cell lines.

Furthermore, our model currently includes only one CDK inhibitor, palbociclib. For a more comprehensive approach testing other CDK inhibitors, our framework could be tailored to evaluate other CDK4/6 inhibitors, such as ribociclib and abemaciclib. To broaden the analysis that we conducted for palbociclib and incorporate other cell-cycle inhibitors, we would require *in vitro* data derived from drug synergy and cell-cycle assays with the other inhibitors. With this data, pharmacodynamic parameters can be estimated, and when combined with pharmacokinetic models obtained from clinical trials of these drugs, we could perform *in silico* trial simulations. This could result in a more inclusive model and would strengthen its applicability, potentially offering insights into the interaction of different cell-cycle checkpoints with various CDK inhibitors. As a relevant example, a selective CDK4 inhibitor (PF-07220060) is currently in clinical development (NCT04557449; ref. 46). PF-07220060 does not inhibit CDK6, which is the main mediator of neutropenia (51), and this may allow higher doses in a continuous fashion that could result in better patient outcomes. In addition, inhibitors of CDK7, a cyclin-activating kinase, CDK2 and CDK2/4/6 inhibitors are in clinical development in ER^+^ breast cancer (52). Thus, although we tested one CDK4/6i, we provide a framework that enables the evaluation of the scheduling of other cell-cycle inhibitors.

In our study, the 95% credible intervals of the posterior predictions were large. This issue might be addressed by an alternative experimental design using the FastFUCCI assay, which may improve both labelling efficiency and expression rate (higher spatial resolution; ref. 53) and/or alternate modeling strategies. For instance, in future work we will consider using stochastic processes to model cell growth directly: with this approach, variations of cell growth become an intrinsic property determined by the stochastic process and the parameters of these variations can be approximated by the Central Limit Theorem for multitype branching processes (54). Data from cell-cycle assays can then be used, together with the estimation tool provided in ref. 54 to determine parameters of a multistage cell-cycle stochastic model.

Finally, by integrating our simulations for the pharmacodynamics of palbociclib and fulvestrant (Fig. 3; Table 2), we found that the use of the conventional metric IC_50_ leads to results that are either significantly underestimated (the sensitivity of the responses to fulvestrant) or obscured (the synergistic effect). However, advantages of using IC_50_s include straightforward computation and the fact that no prior knowledge of the drug effect is needed. In contrast, although measuring TR-50s is based on the drug effect on inhibiting GRs, the measurement can lead to some bias if too many constraints are included in the model. Therefore, we suggest applying both IC_50_s and TR_50_s, thus enabling complementary contributions to a comprehensive view of the pharmacodynamics of drugs that target cell-cycle checkpoints.

## Supplementary Material

Supplementary Fig. S1 Supplementary Fig. S1 shows an illustration of multistage cell-cycle model with m = 4

Supplementary Fig. S2 Supplementary Fig. S2 shows cell proliferation results in -DOX/+DOX cells

Supplementary Fig. S3 Supplementary Fig. S3 shows cell cycle analysis results

Supplementary Fig. S4 Supplementary Fig. S4 shows using LOOIC to determine number of stages m

Supplementary Fig. S5 Supplementary Fig. S5 shows posterior prediction of the total cell count

Supplementary Fig. S6 Supplementary Fig. S6 shows posterior prediction of the cell cycle phase counts

Supplementary Fig. S7 Supplementary Fig. S7 shows posterior prediction of effective drug model parameters

Supplementary Fig. S8 Supplementary Fig. S8 shows response surfaces and contour plots of the drug pairs

Supplementary Fig. S9 Supplementary Fig. S9 shows in silico trial predictions of multiple palbociclib treatment administration schedules in combination with fulvestrant

Supplementary Fig. S10 Supplementary Fig. S10 shows parameter inference of palbociclib resistant cell lines

Supplementary Fig. S11 Supplementary Fig. S11 shows in silico simulation of palbociclib resistant MCF7 cell line

Supplementary Fig. S12 Supplementary Fig. S12 shows parameter search of palbociclib resistant cell lines

Supplementary Fig. S13 Supplementary Fig. S13 shows in silico simulation of new cell lines - 1

Supplementary Fig. S14 Supplementary Fig. S14 shows in silico simulation of new cell lines - 2

Supplementary Fig. S15 Supplementary Fig. S15 shows in silico simulation of new cell lines - 3

Supplementary Fig. S16 Supplementary Fig. S16 shows in silico trial predictions of the cell count at day 100

Supplementary Table S1 Supplementary Table S1 shows parameter distributions for model inference

Supplementary Table S2 Supplementary Table S2 shows quantifications of cell growth rates

Supplementary Table S3 Supplementary Table S3 shows ratio of each schedule to the standard schedule at day 100 for simulated cell lines

Supplementary Appendix S1 Supplementary Appendix S1 shows multistage model

Supplementary Appendix S2 Supplementary Appendix S2 shows Bayesian estimation of model parameters

Supplementary Appendix S3 Supplementary Appendix S3 shows multistage model versus logistic model
