# Supplementary material for: Mathematical Modeling Identifies Optimum Palbociclib-fulvestrant Dose Administration Schedules for the Treatment of Patients with Estrogen Receptor–positive Breast Cancer
Source: Cancer Res Commun. 2023 Nov 16;3(11):2331–44. doi: 10.1158/2767-9764.CRC-23-0257 (PMC10652811; doi:10.1158/2767-9764.CRC-23-0257)

Fig. S1 An illustration of multistage cell-cycle model with $m = 4$. Each filled circle with $x_i,\ i = 1, \cdots, 12$ represents the cell number at each subphase, and $x_1 + x_2 + x_3 + x_4$ gives rise to x_α for the cell number at G0/G1 phase, which is also applied to $x_5 + x_6 + x_7 + x_8 = x_\beta$ for S phase and $x_9 + x_{10} + x_{11} + x_{12} = x_\gamma$ for G2/M phase. The arrows between each subphase represent cell transitions. Within each phase, all subphases share the same transition rate, but the rates can be different among the three phase. There are two key transitions: (1) the transition (red arrow) between x_4 and x_5, from the end of G1/G0 to the first subphase of S, has the transition rate modeled by a function of concentrations of fulvestrant and palbociclib; (2) the transitions (yellow arrows) represent the cell division after completing a cycle. Then both of the divided cells start their own new cycle, separately.

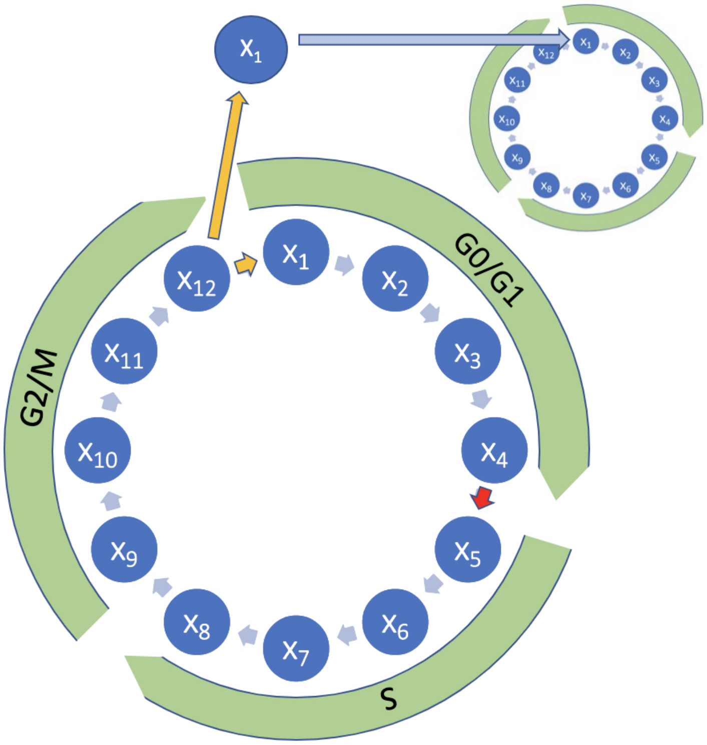

Supplement: Supplementary Fig. S1 — shows an illustration of multistage cell-cycle model with m = 4 [file crc-23-0257-s01.pdf]
