# Supplementary material for: Mathematical Modeling Identifies Optimum Palbociclib-fulvestrant Dose Administration Schedules for the Treatment of Patients with Estrogen Receptor–positive Breast Cancer
Source: Cancer Res Commun. 2023 Nov 16;3(11):2331–44. doi: 10.1158/2767-9764.CRC-23-0257 (PMC10652811; doi:10.1158/2767-9764.CRC-23-0257)

Fig. S2 Cell proliferation results in -DOX/+DOX cells. Total cell growth count in (A) -DOX and (B) +DOX cells. Each panel shows the total number of live cells over five days for a specific combination of palbociclib and fulvestrant. The concentration of fulvestrant increases across the columns and is denoted at the top of each column; the concentration of palbociclib increases down the rows and is denoted to the right of each row. The unit of drug concentrations is nanomolar (nM).

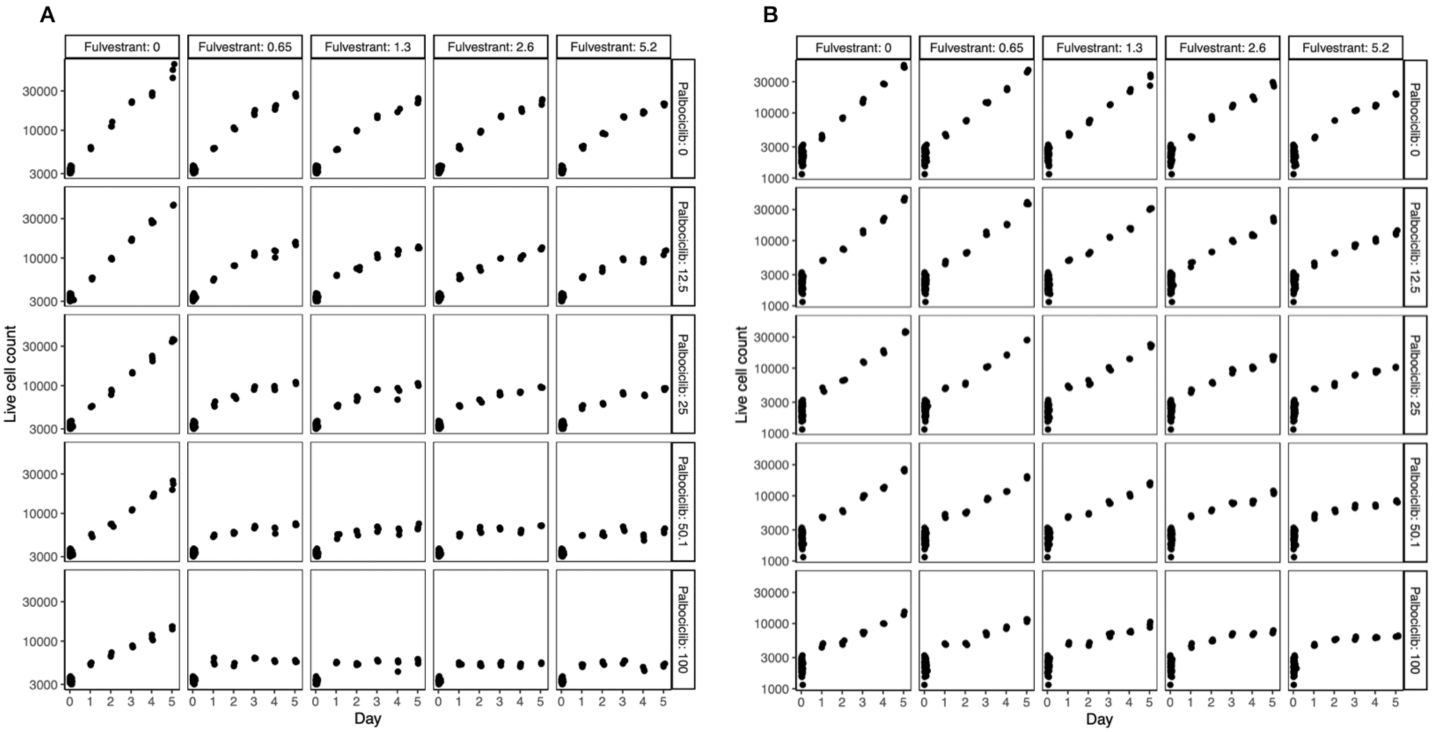

Supplement: Supplementary Fig. S2 — shows cell proliferation results in -DOX/+DOX cells [file crc-23-0257-s02.pdf]
