# Supplementary material for: Mathematical Modeling Identifies Optimum Palbociclib-fulvestrant Dose Administration Schedules for the Treatment of Patients with Estrogen Receptor–positive Breast Cancer
Source: Cancer Res Commun. 2023 Nov 16;3(11):2331–44. doi: 10.1158/2767-9764.CRC-23-0257 (PMC10652811; doi:10.1158/2767-9764.CRC-23-0257)

Fig. S3 Cell cycle analysis results. Distribution of cells in each phase of the cell cycle in (A) -DOX and (B) +DOX cells. The top row in each panel is from the fulvestrant alone treatment, the middle row shows the results from the palbociclib alone treatment, and the bottom row shows the results from the combination treatment. The percentage of cells in G0/G1 is shown in red, S is shown in blue, and G2/M is show in blue. The unit of drug concentrations is nM.

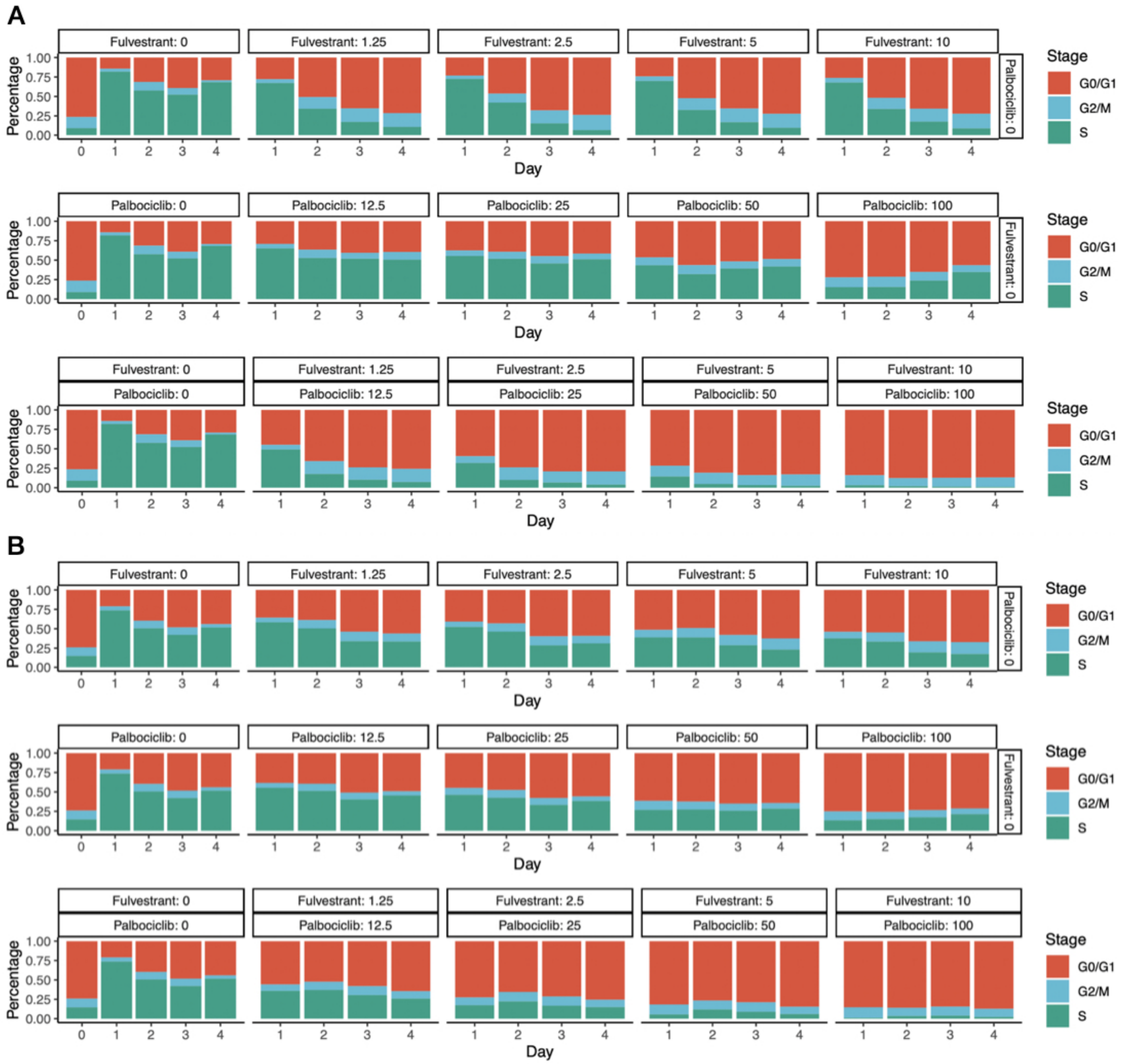

Supplement: Supplementary Fig. S3 — shows cell cycle analysis results [file crc-23-0257-s03.pdf]
