# Supplementary material for: Mathematical Modeling Identifies Optimum Palbociclib-fulvestrant Dose Administration Schedules for the Treatment of Patients with Estrogen Receptor–positive Breast Cancer
Source: Cancer Res Commun. 2023 Nov 16;3(11):2331–44. doi: 10.1158/2767-9764.CRC-23-0257 (PMC10652811; doi:10.1158/2767-9764.CRC-23-0257)

Fig. S4 Using LOOIC to determine number of stages m. LOOIC comparison among different m for (A) -DOX and (B) +DOX cells. The values were rescaled: The best model was set to zero. Having more negative values means the model has worse prediction. (A) shows $m = 8$ is the best model for -DOX and (B) shows $m = 2$ is the best model for +DOX in terms of model predictions. (C) and (D) are the cartoon-like figures for visualizing the most probable number of subphases of each phase for -DOX and +DOX cells, respectively.

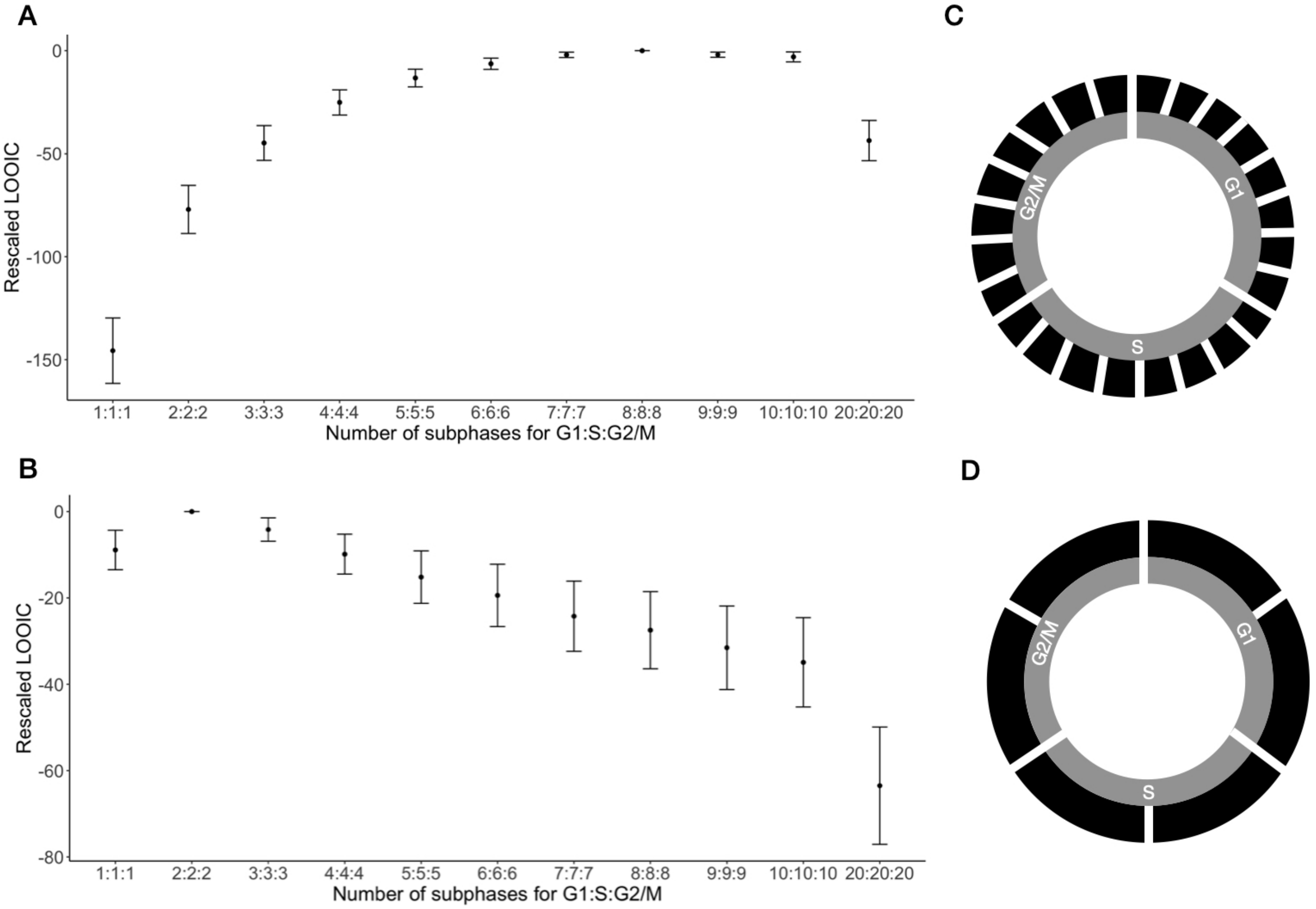

Supplement: Supplementary Fig. S4 — shows using LOOIC to determine number of stages m [file crc-23-0257-s04.pdf]
