# Supplementary material for: Mathematical Modeling Identifies Optimum Palbociclib-fulvestrant Dose Administration Schedules for the Treatment of Patients with Estrogen Receptor–positive Breast Cancer
Source: Cancer Res Commun. 2023 Nov 16;3(11):2331–44. doi: 10.1158/2767-9764.CRC-23-0257 (PMC10652811; doi:10.1158/2767-9764.CRC-23-0257)

Fig. S5 Posterior prediction of the total cell count. Each panel shows the posterior predicted total number of live cells over five days for a specific combination of palbociclib and fulvestrant for (A) -DOX and (B) +DOX cells. The line represents the median posterior predicted live cell count value over five days and the gray shaded area corresponds to the 95% credible interval of the posterior predictive values. The data points represent the observed cell counts from the drug synergy experiments used to train the model. The concentration of palbociclib increases across the columns and is denoted at the top of each column; the concentration of fulvestrant increases down the rows and is denoted to the right of each row. The unit of drug concentrations is nM. The average of the median cell counts over all panels is 10523 for -DOX cells and 11281 for +DOX cells, but the average of 95% credible intervals is 3973 for -DOX cells and 4554 for +DOX cells, i.e., the 95% credible intervals only span $1/2 - 1/3$ orders of magnitude of the medians.

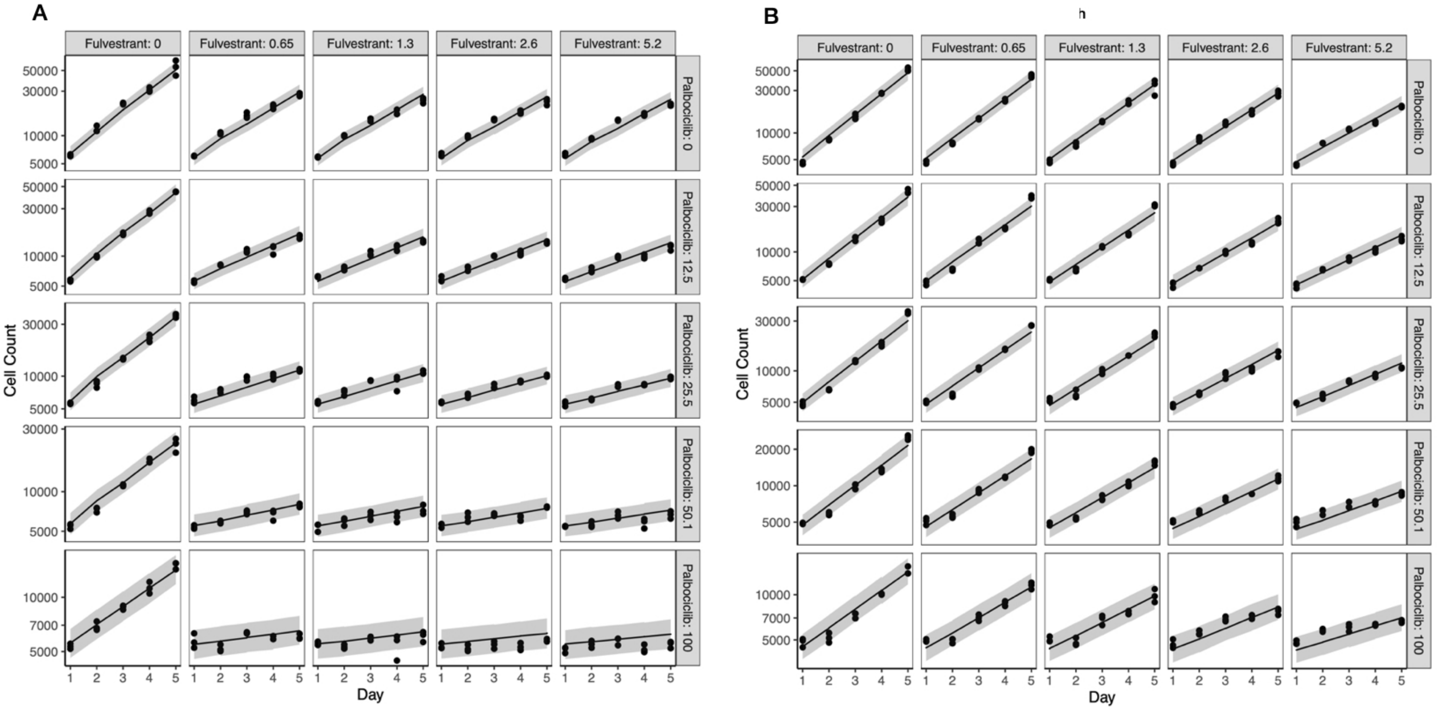

Supplement: Supplementary Fig. S5 — shows posterior prediction of the total cell count [file crc-23-0257-s05.pdf]
