# Supplementary material for: Mathematical Modeling Identifies Optimum Palbociclib-fulvestrant Dose Administration Schedules for the Treatment of Patients with Estrogen Receptor–positive Breast Cancer
Source: Cancer Res Commun. 2023 Nov 16;3(11):2331–44. doi: 10.1158/2767-9764.CRC-23-0257 (PMC10652811; doi:10.1158/2767-9764.CRC-23-0257)

Fig. S6 Posterior prediction of the cell cycle phase counts. The posterior predictive number of cells in each phase of the cell cycle for (A) -DOX (B) +DOX cells treated with (1) fulvestrant only, (2) palbociclib only, or (3) fulvestrant and palbociclib in combination. Each panel corresponds to a specific dose of fulvestrant and/or palbociclib. The line represents the median posterior predictive value and the shaded area corresponds to the 95% credible interval of the posterior predictive values. The datapoints represent the observed cell phase counts from the cell cycle analysis experiments used to train the model. The concentrations of fulvestrant and palbociclib increase across the columns and are denoted at the top of each column. Red, blue, and green represent the G0/G1, S, and G/M phases, respectively. The average of median cell counts over all panels is 2210 for -DOX cells and 1989 for +DOX cells, and the average of 95% credible intervals is 7737 for -DOX cells and 4754 for +DOX cells. This result showed that the 95% credible intervals often span two or three orders of magnitude of the medians.

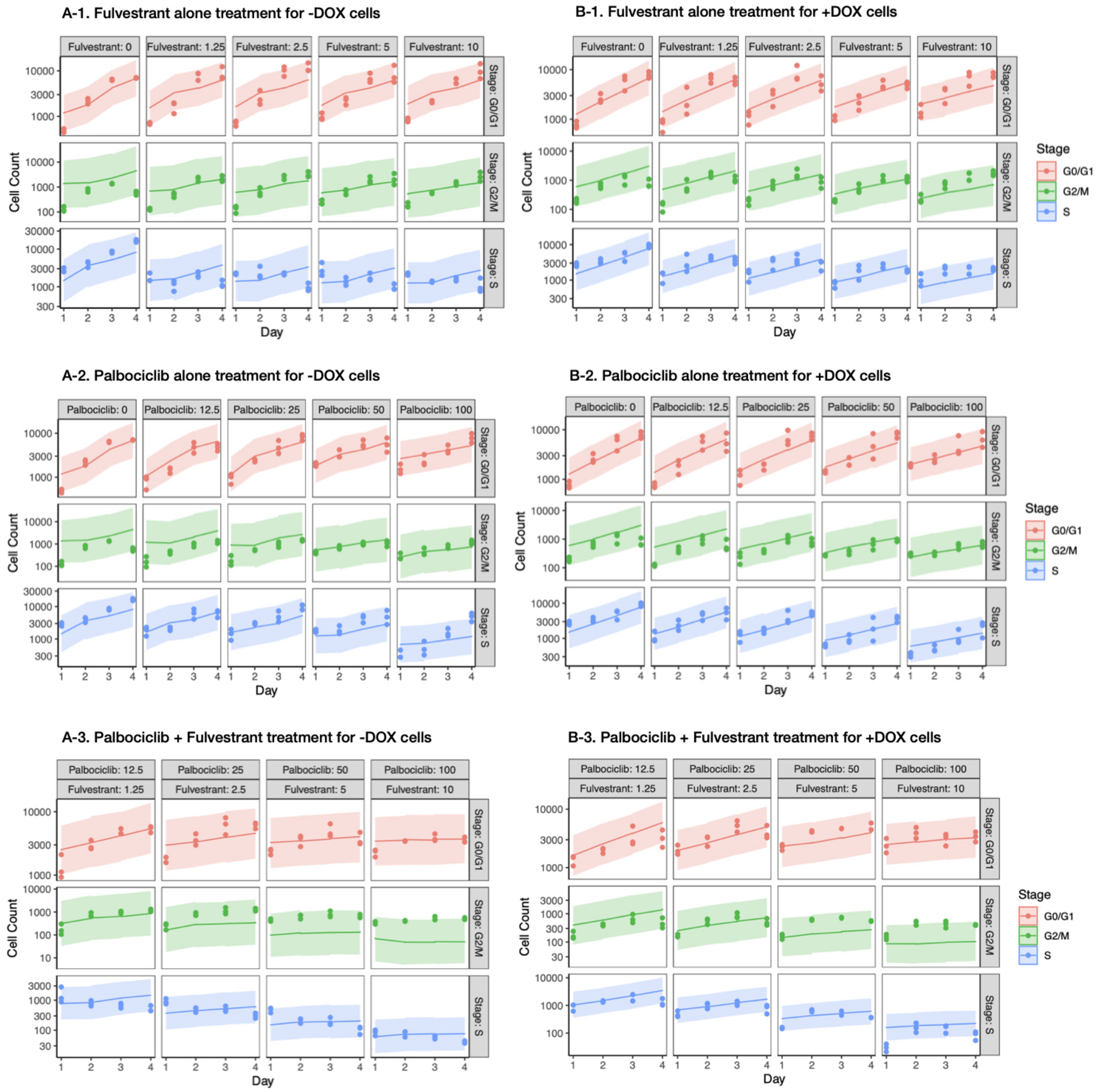

Supplement: Supplementary Fig. S6 — shows posterior prediction of the cell cycle phase counts [file crc-23-0257-s06.pdf]
