# Supplementary material for: Mathematical Modeling Identifies Optimum Palbociclib-fulvestrant Dose Administration Schedules for the Treatment of Patients with Estrogen Receptor–positive Breast Cancer
Source: Cancer Res Commun. 2023 Nov 16;3(11):2331–44. doi: 10.1158/2767-9764.CRC-23-0257 (PMC10652811; doi:10.1158/2767-9764.CRC-23-0257)

Fig. S7 Posterior prediction of effective drug model parameters. (A) - (D) In each panel, the y-axis represents the density of the model parameter given by 3000 samples from the posterior distributions, and the x-axis represents the values of G1/S-TR50 in the unit of nM. (A) fulvestrant for -DOX cells (B) palbociclib for -DOX cells (D)fulvestrant for -DOX cells (E) palbociclib for +DOX cells. (E) and (F) are the densities for the interaction parameter a_{FP}. The vertical lines indicate the zero value of the parameter and the area of the black region is 12.9% of the total area for both (E) and (F).

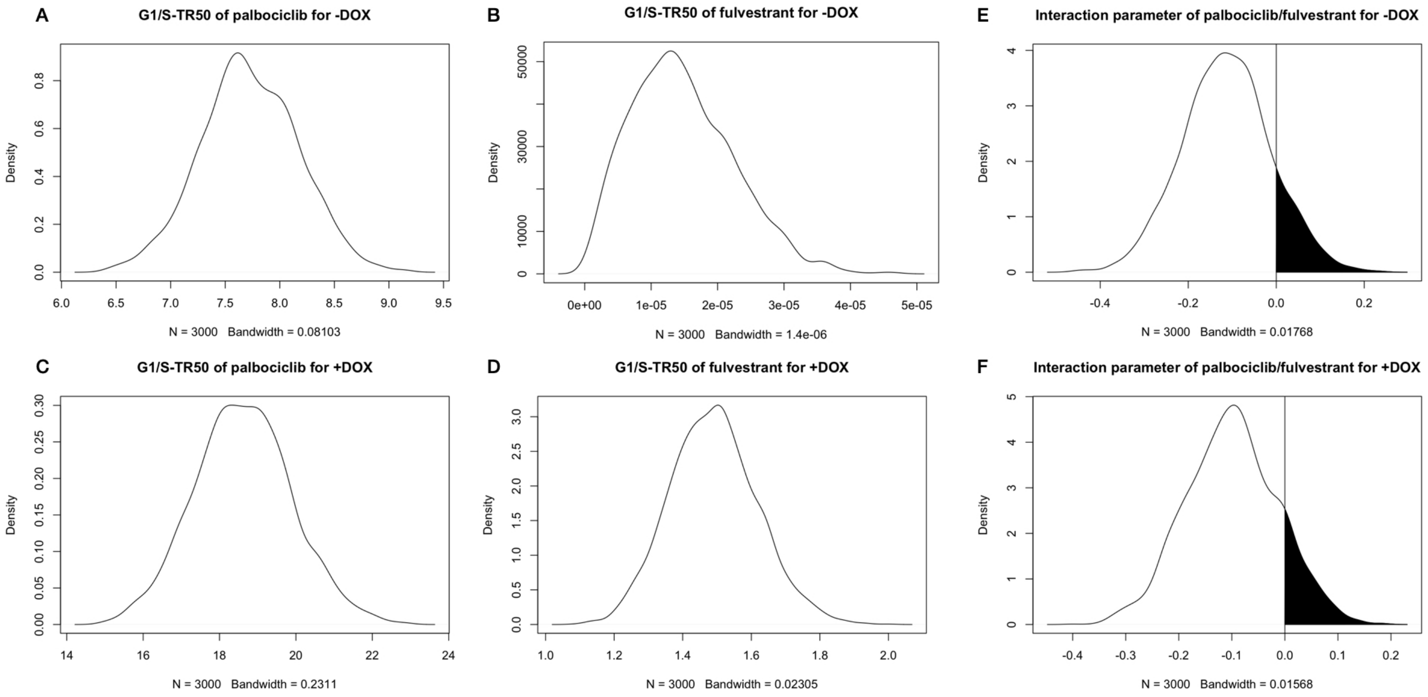

Supplement: Supplementary Fig. S7 — shows posterior prediction of effective drug model parameters [file crc-23-0257-s07.pdf]
