# Supplementary material for: Mathematical Modeling Identifies Optimum Palbociclib-fulvestrant Dose Administration Schedules for the Treatment of Patients with Estrogen Receptor–positive Breast Cancer
Source: Cancer Res Commun. 2023 Nov 16;3(11):2331–44. doi: 10.1158/2767-9764.CRC-23-0257 (PMC10652811; doi:10.1158/2767-9764.CRC-23-0257)

Fig. S8 Response surfaces and contour plots of the drug pairs. (A) and (C) are the surface plots for G1/S-TR50 (z-axis) with respect to the combinations of palbociclib (x-axis) and fulvestrant (y-axis). (A) for -DOX cells: since the response to fulvestrant was extremely sensitive, the unit was rescaled to $1e^{-6}$ nM; palbocilib was in the unit of nM. (C) for +DOX cells: the units of palbociclib and fulvestrant were both in nM. (B) and (D) are the contour plots corresponding to (A) and (C), respectively.

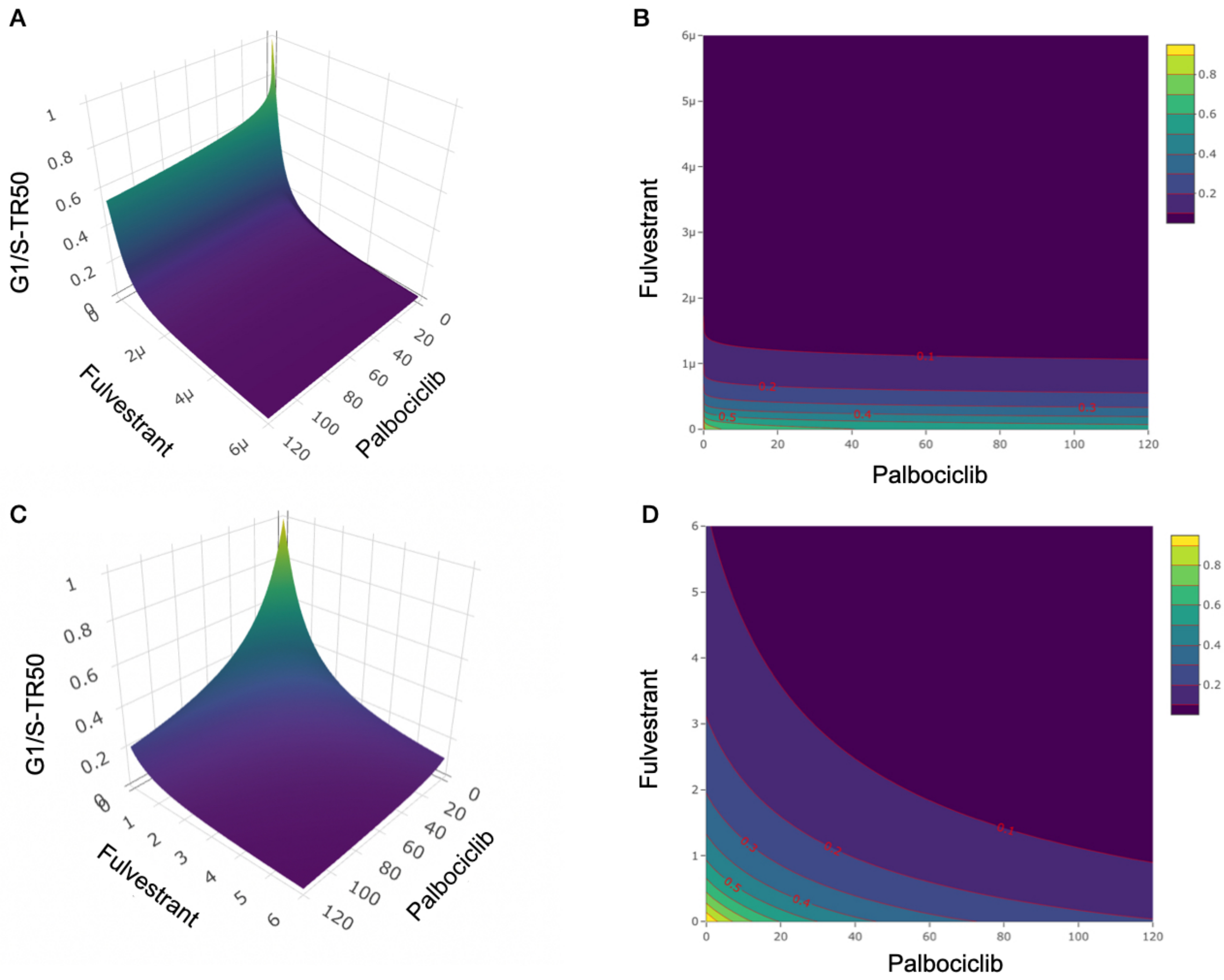

Supplement: Supplementary Fig. S8 — shows response surfaces and contour plots of the drug pairs [file crc-23-0257-s08.pdf]
