# Supplementary material for: Mathematical Modeling Identifies Optimum Palbociclib-fulvestrant Dose Administration Schedules for the Treatment of Patients with Estrogen Receptor–positive Breast Cancer
Source: Cancer Res Commun. 2023 Nov 16;3(11):2331–44. doi: 10.1158/2767-9764.CRC-23-0257 (PMC10652811; doi:10.1158/2767-9764.CRC-23-0257)

Fig. S9 ***In silico*** **trial predictions of multiple palbociclib treatment administration schedules in combination with fulvestrant.** Each panel represents the plasma concentration of (A) fulvestrant and (B) palbociclib for each treatments schedule given by the table.

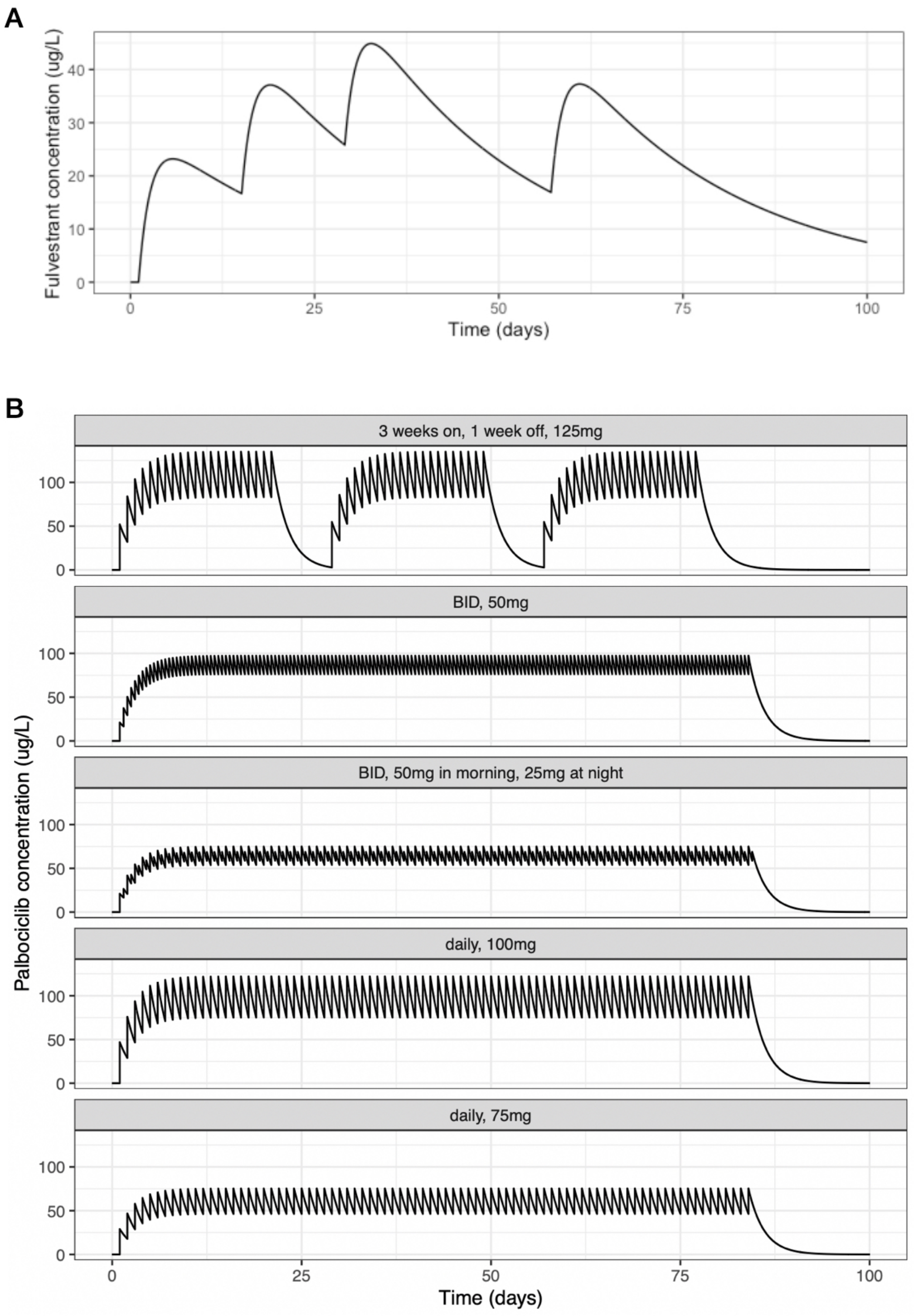

Supplement: Supplementary Fig. S9 — shows in silico trial predictions of multiple palbociclib treatment administration schedules in combination with fulvestrant [file crc-23-0257-s09.pdf]
