# Supplementary material for: Mathematical Modeling Identifies Optimum Palbociclib-fulvestrant Dose Administration Schedules for the Treatment of Patients with Estrogen Receptor–positive Breast Cancer
Source: Cancer Res Commun. 2023 Nov 16;3(11):2331–44. doi: 10.1158/2767-9764.CRC-23-0257 (PMC10652811; doi:10.1158/2767-9764.CRC-23-0257)

Fig. S10 Parameter inference of palbociclib resistant cell lines. (A) To develop Palbociclib-resistant cell models, the ER-WT (-DOX) and ER-mutant (+DOX) MCF7 cells were long-term cultured with escalating doses of palbociclib up to the target dose of 1μM. Cells were defined as resistant (+PR cells) when the ability to proliferate in the presence of palbociclib 1 μM was restored. As a control, we long-term cultured the ER-WT and ER-mutant MCF7 cells with the vehicle of the drug (dimethyl sulfoxide, DMSO). (B) The line represents the median posterior predicted live cell count value over six days and the gray shaded area corresponds to the 95% credible interval of the posterior predictive values. The data points represent the observed cell counts from the experiment, described in (A), used to train the model. The concentration of palbociclib increases across the columns and is denoted at the top of each column; the concentration of fulvestrant is zero. The unit of drug concentrations is nM.

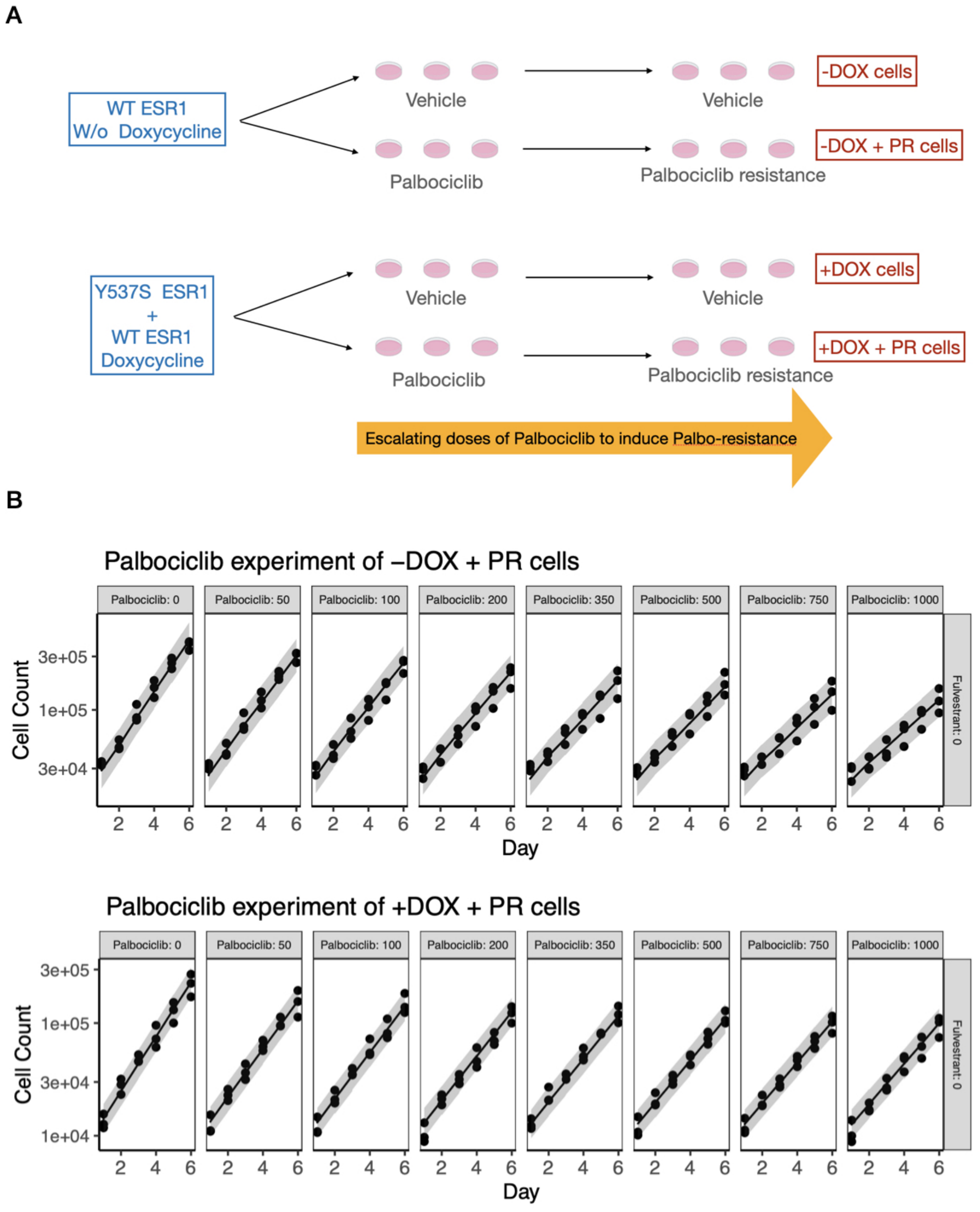

Supplement: Supplementary Fig. S10 — shows parameter inference of palbociclib resistant cell lines [file crc-23-0257-s10.pdf]
