# Supplementary material for: Mathematical Modeling Identifies Optimum Palbociclib-fulvestrant Dose Administration Schedules for the Treatment of Patients with Estrogen Receptor–positive Breast Cancer
Source: Cancer Res Commun. 2023 Nov 16;3(11):2331–44. doi: 10.1158/2767-9764.CRC-23-0257 (PMC10652811; doi:10.1158/2767-9764.CRC-23-0257)
Supplement: Supplementary Fig. S11 — shows in silico simulation of palbociclib resistant MCF7 cell line [file crc-23-0257-s11.pdf]

Fig. S11 ***In silico* simulation of palbociclib resistant MCF7 cell line.** By the *in silico* trial predictions of multiple palbociclib treatment administration schedules in combination with fulvestrant, (A) - (D) are the predictions of cell growth trajectories by taking the ratio of each schedule to the standard schedule (daily, 125mg, 3w on, 1w off). The shaded area corresponds to the 95% credible interval of the posterior predictive values. (A) and (B) are the predictions for the original -DOX and +DOX MCF7 cell lines. (C) and (D) are the predictions for the -DOX and +DOX palbociclib resistant (+PR) MCF7 cell lines. (E) is a table showing the ratio between the predicted number of cells at day 100 for each proposed treatment schedule and the standard pulsed treatment schedule. In addition, the difference between the proposed schedule and the standard schedule was tested for statistical significance using the Wilcoxon test, and the corresponding p-values are reported.

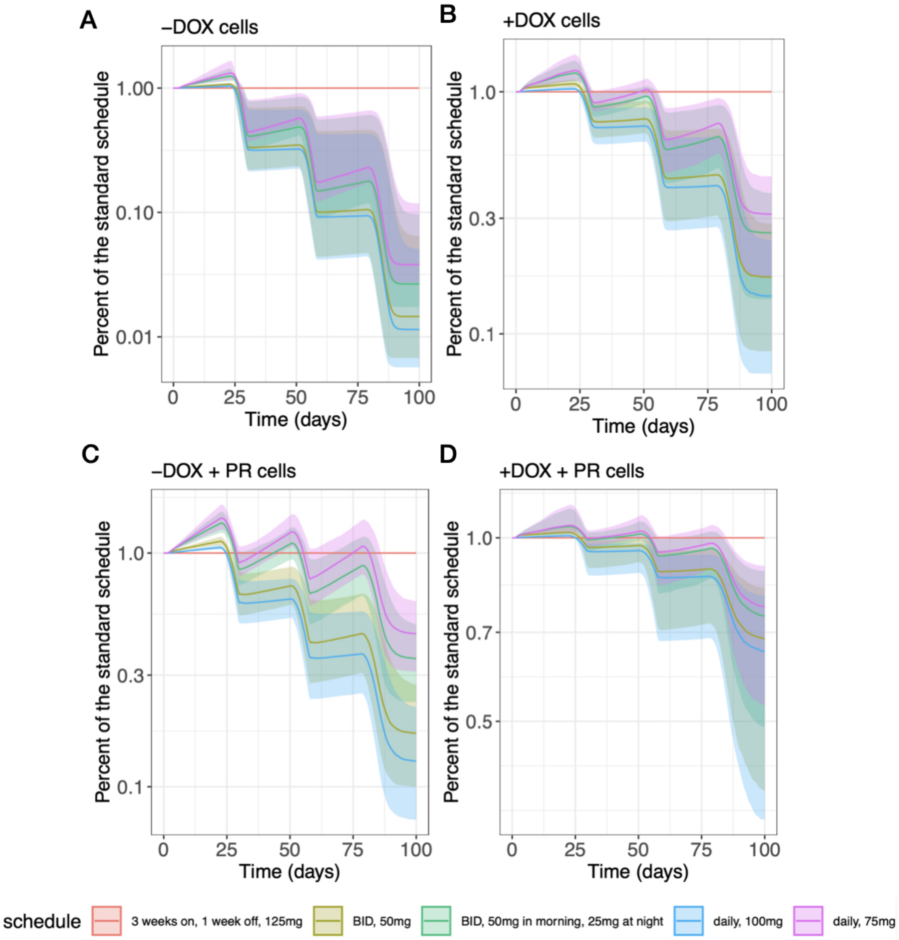

E

The ratio of each schedule to the standard scheulde at day 100 (with p-values)

	-DOX	+DOX	-DOX + PR	+DOX + PR
**Daily, 100mg**	0.0115 (p < 2.22e-16)	0.1434 (p < 2.22e-16)	0.1289 (p < 2.22e-16)	0.6515 (p = 0.028)
**BID, 50/50mg**	0.0146 (p < 2.22e-16)	0.1715 (p < 2.22e-16)	0.1695 (p < 2.22e-16)	0.6841 (p = 0.051)
**Daily, 75mg**	0.038 (p < 2.22e-16)	0.312 (p < 2.22e-16)	0.4524 (p = 1.1e-13)	0.7721 (p = 0.18)
**BID, 50/25mg**	0.0266 (p < 2.22e-16)	0.2613 (p < 2.22e-16)	0.3533 (p < 2.22e-16)	0.7454 (p = 0.13)
