# Supplementary material for: Mathematical Modeling Identifies Optimum Palbociclib-fulvestrant Dose Administration Schedules for the Treatment of Patients with Estrogen Receptor–positive Breast Cancer
Source: Cancer Res Commun. 2023 Nov 16;3(11):2331–44. doi: 10.1158/2767-9764.CRC-23-0257 (PMC10652811; doi:10.1158/2767-9764.CRC-23-0257)

Fig. S12 Parameter search. Each panel (A) -DOX cells with palbociclib resistance (B) +DOX cells with palbociclib resistance shows the total number of cells treated by a specific concentration of palbociclib (from the top down: 0, 50.1, 100 nM) and zero fulvestrant. Black dots in left panel are three replicates of -DOX+PR cells and in right panel are three replicates of +DOX+PR cells over five days. The blue curves and red curves are the simulations of -DOX+PR cell and +DOX+PR cell growth, respectively, based on our parameter grid search: we varied the original parameters of -DOX/+DOX cells to simulate the cases when the -DOX/+DOX cells acquired palbociclib resistance. The results validate the range of adjusted parameters are relevant to the palbociclib resistance.

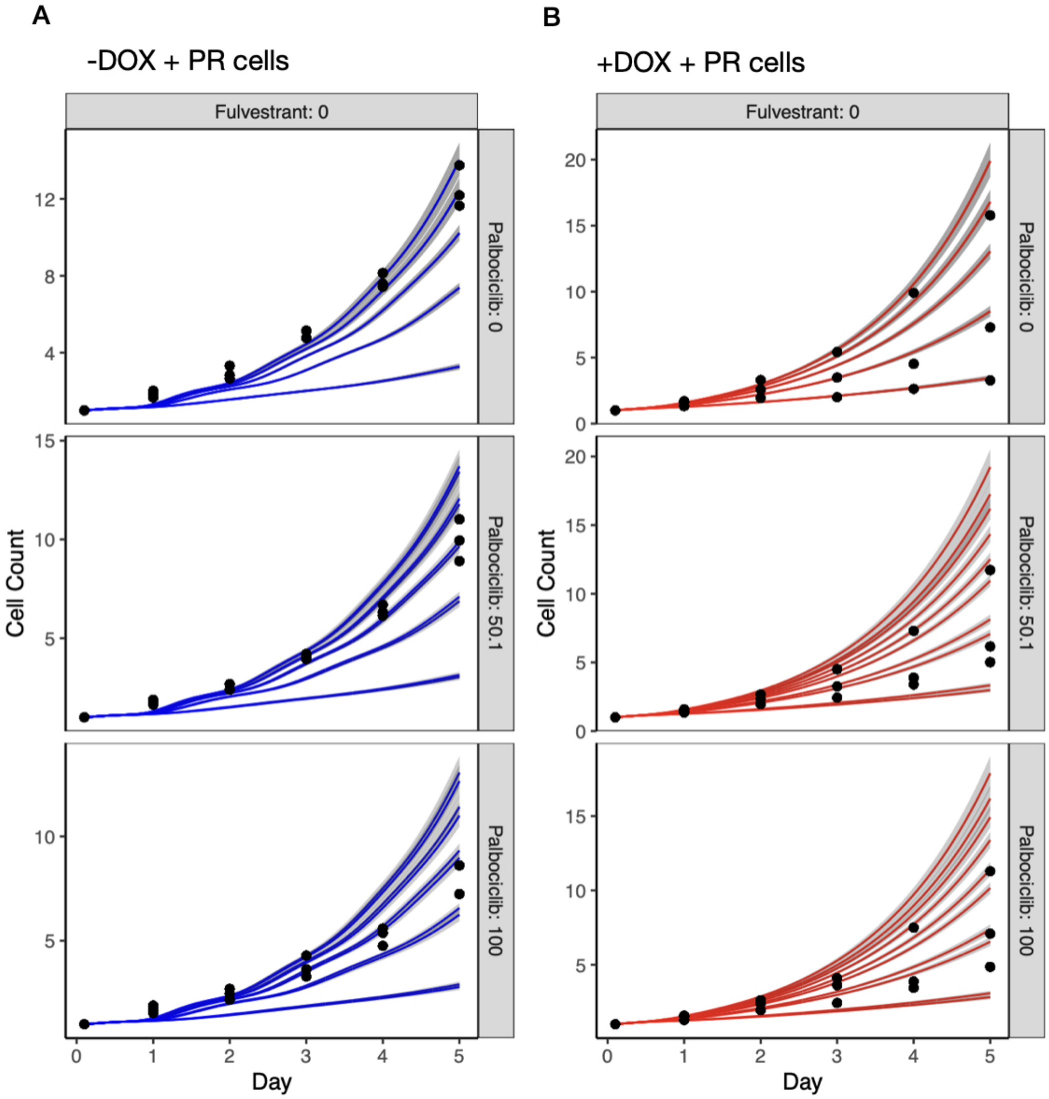

Supplement: Supplementary Fig. S12 — shows parameter search of palbociclib resistant cell lines [file crc-23-0257-s12.pdf]
