# Supplementary material for: Mathematical Modeling Identifies Optimum Palbociclib-fulvestrant Dose Administration Schedules for the Treatment of Patients with Estrogen Receptor–positive Breast Cancer
Source: Cancer Res Commun. 2023 Nov 16;3(11):2331–44. doi: 10.1158/2767-9764.CRC-23-0257 (PMC10652811; doi:10.1158/2767-9764.CRC-23-0257)

Fig. S13 ***In silico*** **simulation of new cell lines - 1** (A) for -DOX cells and (B) for +DOX cells are the standard box plots for the number of cells at day 100. From left to right, we varied the untreated G1-S transition from the original cell line (MCF7) by giving different weights (0.2, 0.6, 1.0, 1.4, 2.0) of the original parameter λ_{α}. In x-axis, G denotes the weight of G1-S growth rate, B denotes the weight of the slope of palbocicilib response curve, and C denotes the weight of the half maximal inhibitory concentration (G1/S-TR50) of palbocicilib response curve.

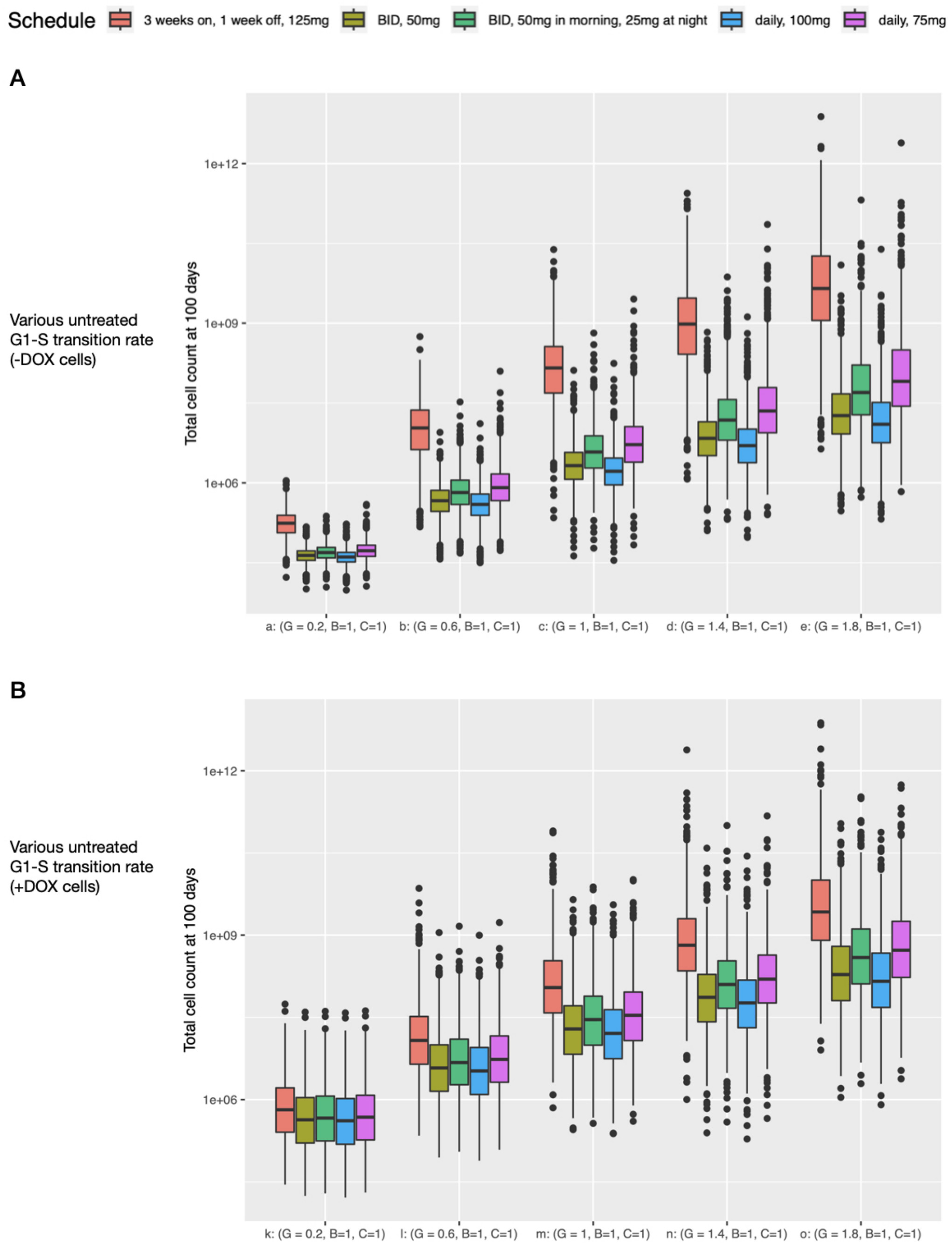

Supplement: Supplementary Fig. S13 — shows in silico simulation of new cell lines - 1 [file crc-23-0257-s13.pdf]
