# Supplementary material for: Mathematical Modeling Identifies Optimum Palbociclib-fulvestrant Dose Administration Schedules for the Treatment of Patients with Estrogen Receptor–positive Breast Cancer
Source: Cancer Res Commun. 2023 Nov 16;3(11):2331–44. doi: 10.1158/2767-9764.CRC-23-0257 (PMC10652811; doi:10.1158/2767-9764.CRC-23-0257)

Fig. S14 ***In silico*** **simulation of new cell lines - 2** (A) for -DOX cells and (B) for +DOX cells are the standard box plots for the number of cells at day 100. From left to right, we varied the EC50s of palbocicilib response curve from the original cell line (MCF7) by giving different weights (0.01, 0.1, 1.0, 10, 100) of the original parameter c_P. In x-axis, G denotes the weight of G1-S growth rate, B denotes the weight of the slope of palbocicilib response curve, and C denotes the weight of G1/S-TR50 of palbocicilib response curve.

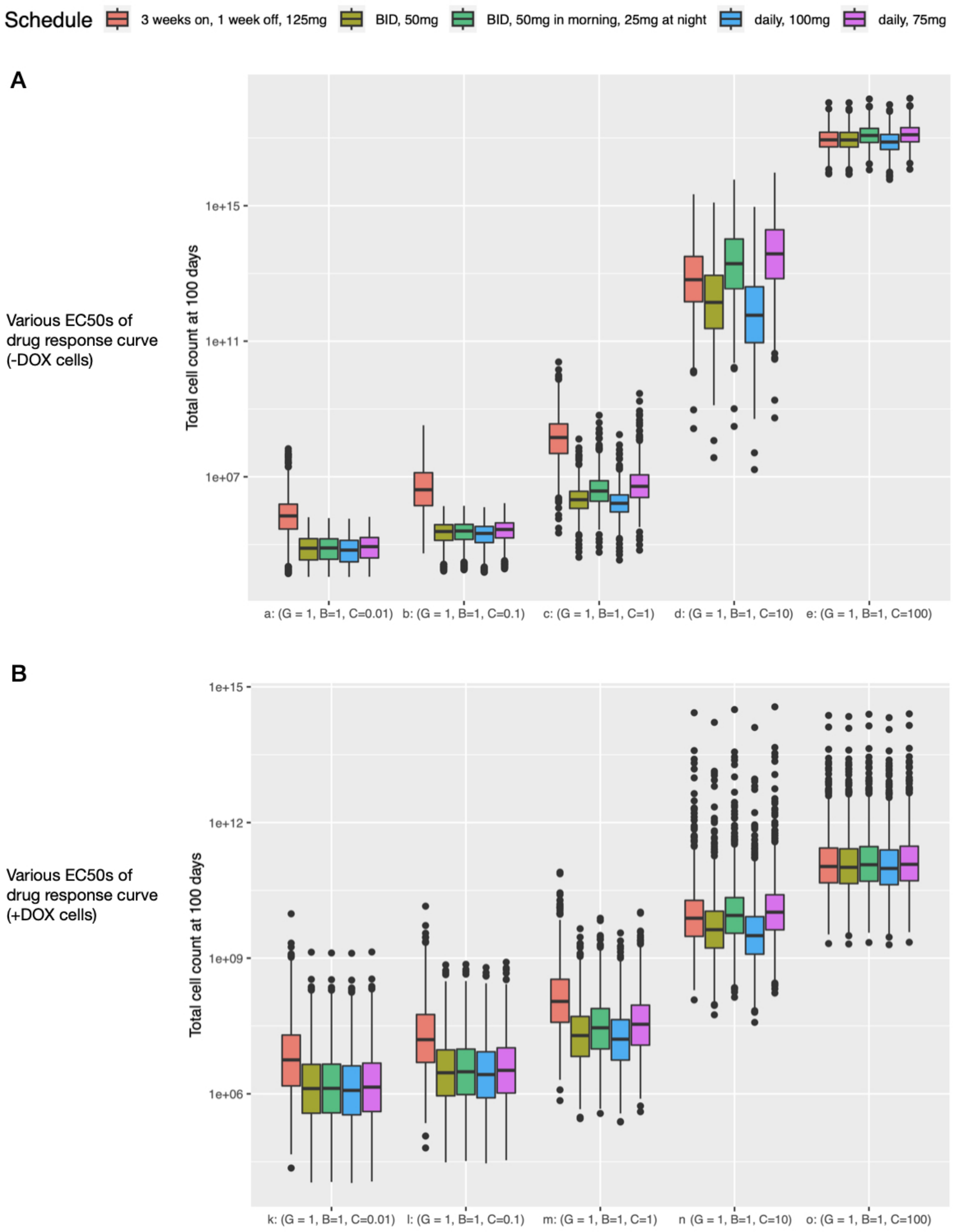

Supplement: Supplementary Fig. S14 — shows in silico simulation of new cell lines - 2 [file crc-23-0257-s14.pdf]
