# Supplementary material for: Mathematical Modeling Identifies Optimum Palbociclib-fulvestrant Dose Administration Schedules for the Treatment of Patients with Estrogen Receptor–positive Breast Cancer
Source: Cancer Res Commun. 2023 Nov 16;3(11):2331–44. doi: 10.1158/2767-9764.CRC-23-0257 (PMC10652811; doi:10.1158/2767-9764.CRC-23-0257)

Fig. S16 ***In silico* trial predictions of the cell count at day 100 with initial tumor heterogeneity.** (A) for the original cell line (MCF7) (B) for a simulated cell line with a high untreated G1-S transition rate ($1.8 * \lambda_\alpha$) (C) for a simulate cell line with high palbociclib resistance ($10 * c_P$). (A)-(C) are the standard box plots for the number of cells at day 100 with different degrees of initial tumor heterogeneity at day 0. From left to right, a: 100% -DOX cells, b: 80% -DOX cells + 20% +DOX cells, c: 50% -DOX cells + 50% +DOX cells, d: 20% -DOX cells + 80% +DOX cells, e: 100% +DOX cells.

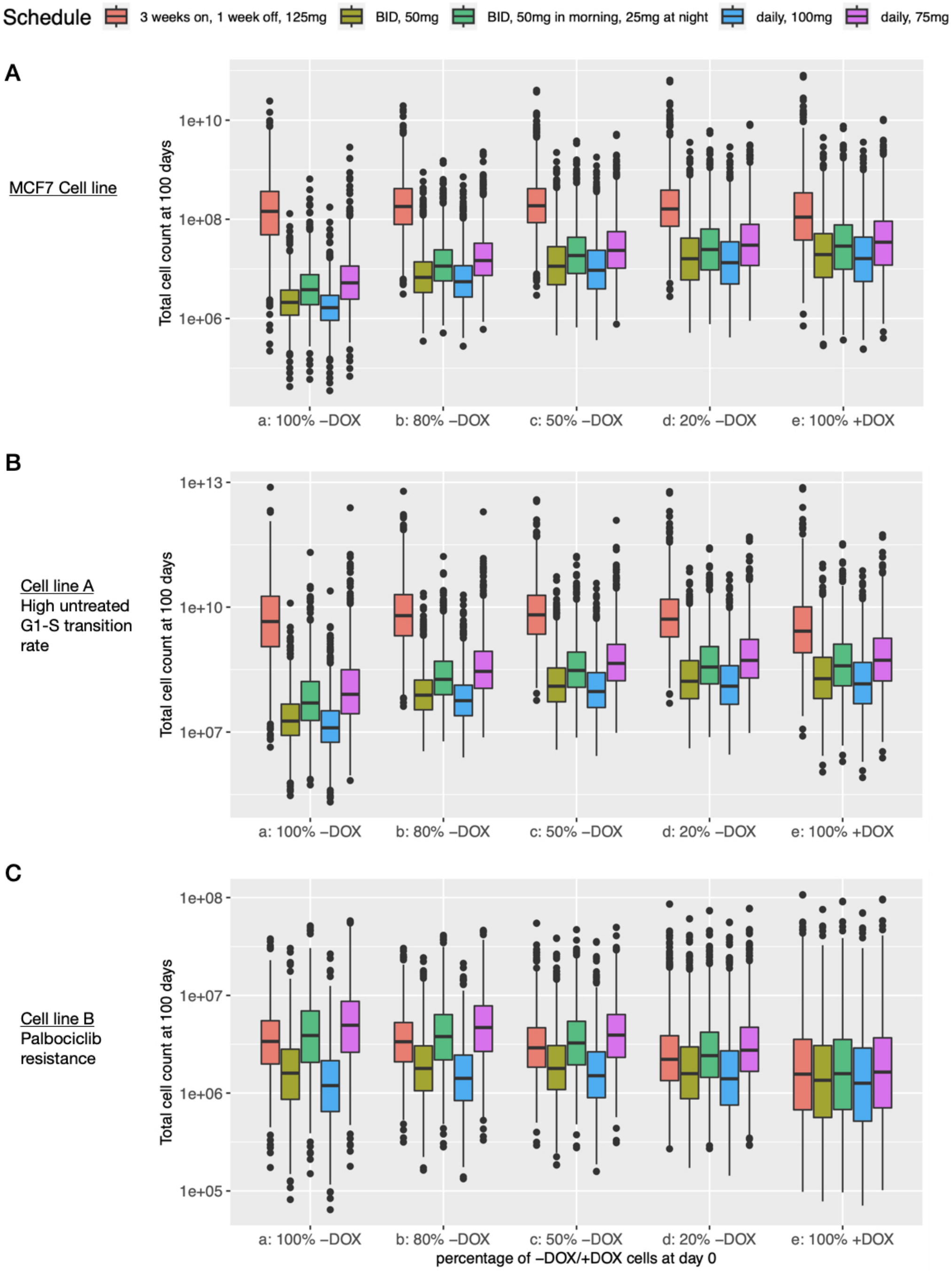

Supplement: Supplementary Fig. S16 — shows in silico trial predictions of the cell count at day 100 [file crc-23-0257-s16.pdf]
