# Supplementary material for: Mathematical Modeling Identifies Optimum Palbociclib-fulvestrant Dose Administration Schedules for the Treatment of Patients with Estrogen Receptor–positive Breast Cancer
Source: Cancer Res Commun. 2023 Nov 16;3(11):2331–44. doi: 10.1158/2767-9764.CRC-23-0257 (PMC10652811; doi:10.1158/2767-9764.CRC-23-0257)
Supplement: Supplementary Table S1 — shows parameter distributions for model inference [file crc-23-0257-s17.pdf]

Table. S1 Parameter distributions for model inference. All rates are in day^{-1}; the unit of fulvestrant doses (i.e c_F) is 10^{-5} nM for -DOX cells and nM for +DOX cells; the unit of palbociclib doses (i.e. c_P) is 10 nM. Furthermore, some parameters in the effective drug dose model are rescaled in the following way: b_F is rescaled by $0.1b_F$ in the model of -DOX cells; $\lambda_\alpha^{(max)}$ is rescaled by $0.1\lambda_\alpha^{(max)}$ in the model of -DOX cells; the interaction parameter a_{FP} is rescaled by $0.1a_{FP}$ in the model of both -DOX and +DOX cells. Note that para. is a short notation of parameter and *lnorm* represents the Log-normal distribution.

**Para.**	**Description**	**Prior Distribution**		**Lower bound**	**Upper bound**	**Initial distribution**	
λ_α	G0/G1 subphases transition rate and G0/G1 to S rate (no drug)	$N(16,2)$ for -DOX	$N(4,2)$ for +DOX	0	∞	$lnorm(1, log(16), 1)$ for -DOX	$lnorm(1, log(4), 1)$ for +DOX
$\lambda_\alpha^{(max)}$	G0/G1 to S rate (infinite drug)	$N(1,1)$		0	∞	$lnorm(1, log(0.1), 0.1)$	
λ_β	S subphases transition rate and S to G2/M rate	$N(16,2)$ for -DOX	$N(4,2)$ for +DOX	0	∞	$lnorm(1, log(16), 1)$ for -DOX	$lnorm(1, log(4), 1)$ for +DOX
λ_γ	G2/M subphases transition rate and G2/M to G0/G1 rate	$N(16,2)$ for -DOX	$N(4,2)$ for +DOX	0	∞	$lnorm(1, log(16), 1)$ for -DOX	$lnorm(1, log(4), 1)$ for +DOX
b_P	Palbociclib response steepness	$N(1,1)$		0	∞	$lnorm(1, log(1), 1)$	
b_F	Fulvestrant response steepness	$N(1,1)$		0	∞	$lnorm(1, log(1), 1)$	
c_P	50% effect dose of palbociclib	$N(1,1)$		0	∞	$lnorm(1, log(1), 1)$	
c_F	50% effect dose of fulvestrant	$N(1,1.0)$ for -DOX	$N(1,0.5)$ for +DOX	0	∞	$lnorm(1, log(1), 1)$	
a_{FP}	50% Drug interaction	$N(-1,1)$		$-\infty$	∞	$-lnorm(1, log(1), 1)$	
σ_α	G0/G1 sampling variance	$cauchy(0,1)$		0	∞	$abs(N(1,0.1))$	
σ_β	S sampling variance	$cauchy(0,1)$		0	∞	$abs(N(1,0.1))$	
σ_γ	G2/M sampling variance	$cauchy(0,1)$		0	∞	$abs(N(1,0.1))$	
σ_T	Total count sampling variance	$cauchy(0,1)$		0	∞	$abs(N(1,0.1))$	
