# Supplementary material for: Mathematical Modeling Identifies Optimum Palbociclib-fulvestrant Dose Administration Schedules for the Treatment of Patients with Estrogen Receptor–positive Breast Cancer
Source: Cancer Res Commun. 2023 Nov 16;3(11):2331–44. doi: 10.1158/2767-9764.CRC-23-0257 (PMC10652811; doi:10.1158/2767-9764.CRC-23-0257)
Supplement: Supplementary Table S2 — shows quantifications of cell growth rates [file crc-23-0257-s18.pdf]

Table. S2 Quantifications of cell growth rates. Linear regression method was applied to the log transformation of the *in vitro* dataset (Fig. S2). The slope from the regression coefficients are listed in the table to quantity the cell growth rates. The unit is log of total cell count per day.

For -DOX cells, we find the growth rate decreases significantly according to the increase of either the concentration of palbociclib (P) or the concentration of fulvestrant (F). This result supports our observation in Fig. S2 that cell growth was inhibited when the dose of either palbociclib or fulvestrant increases in -DOX cells. To compare +DOX cells with -DOX cells, by the difference between the two columns (F: 0nm and F: 0.65nm), we found that the difference in table 2 (+DOX cells) is significantly smaller than the difference in table 1 (-DOX cells), which confirms our observation in Fig. S2 that low dose fulvestrant is less effective in +DOX cells compared to -DOX cells.

-DOX Cells	F: 0 nm	F: 0.65 nm	F: 1.3 nm	F: 2.6 nm	F: 5.2 nm
P: 0 nm	0.5613	0.4531	0.4233	0.4193	0.406
P: 12.5 nm	0.5252	0.3305	0.3176	0.2997	0.2796
P: 25 nm	0.4697	0.2724	0.2527	0.2375	0.2257
P: 50.1 nm	0.3895	0.1807	0.1691	0.1766	0.146
P: 100 nm	0.3039	0.1426	0.1318	0.119	0.1106

+DOX Cells	F: 0 nm	F: 0.65 nm	F: 1.3 nm	F: 2.6 nm	F: 5.2 nm
P: 0 nm	0.6244	0.5957	0.5583	0.5257	0.4577
P: 12.5 nm	0.5831	0.5502	0.514	0.454	0.3882
P: 25 nm	0.5468	0.4963	0.4661	0.3967	0.3447
P: 50.1 nm	0.4723	0.4325	0.3923	0.3496	0.3006
P: 100 nm	0.377	0.3411	0.3127	0.2886	0.2565
