# Supplementary material for: Mathematical Modeling Identifies Optimum Palbociclib-fulvestrant Dose Administration Schedules for the Treatment of Patients with Estrogen Receptor–positive Breast Cancer
Source: Cancer Res Commun. 2023 Nov 16;3(11):2331–44. doi: 10.1158/2767-9764.CRC-23-0257 (PMC10652811; doi:10.1158/2767-9764.CRC-23-0257)
Supplement: Supplementary Table S3 — shows ratio of each schedule to the standard schedule at day 100 for simulated cell lines [file crc-23-0257-s19.pdf]

Table. S3 Ratio of each schedule to the standard schedule at day 100 for simulated cell lines. Those are tables showing the ratio between the predicted number of cells at day 100 for each proposed treatment schedule and the standard pulsed treatment schedule. In addition, the difference between the proposed schedule and the standard schedule was tested for statistical significance using the Wilcoxon test, and the corresponding p-values are reported. Each column is for a different weight of cp, which is the G1-S TR50 inferred from the palbociclib sensitive MCF7 cells. Those different weights of cp are to simulate different levels of palbociclib resistance in terms of the half maximal inhibitory concentration (G1-S TR 50) in the palbociclib response curve.

-DOX Cells	0.01*cp	0.1*cp	1*cp	10*cp	100*cp
Daily, 100mg	0.0991 (p < 2.22e-16)	0.0532 (p < 2.22e-16)	0.0115 (p < 2.22e-16)	0.0849 (p < 2.22e-16)	0.8604 (p = 7.1e-09)
BID, 50/50mg	0.1124 (p < 2.22e-16)	0.0608 (p < 2.22e-16)	0.0147 (p < 2.22e-16)	0.2062 (p < 2.22e-16)	0.9941 (p = 0.7)
Daily, 75mg	0.1249 (p < 2.22e-16)	0.0697 (p < 2.22e-16)	0.0384 (p < 2.22e-16)	5.3017 (p < 2.22e-16)	1.368 (p < 2.22e-16)
BID, 50/25mg	0.1139 (p < 2.22e-16)	0.063 (p < 2.22e-16)	0.0265 (p < 2.22e-16)	2.801 (p < 2.22e-16)	1.3079 (p < 2.22e-16)

+DOX Cells	0.01*cp	0.1*cp	1*cp	10*cp	100*cp
Daily, 100mg	0.2265 (p < 2.22e-16)	0.1766 (p < 2.22e-16)	0.143 (p < 2.22e-16)	0.4206 (p < 2.22e-16)	0.9098 (p = 0.044)
BID, 50/50mg	0.2475 (p < 2.22e-16)	0.1961 (p < 2.22e-16)	0.1712 (p < 2.22e-16)	0.564 (p < 2.22e-16)	0.9626 (p = 0.4)
Daily, 75mg	0.2657 (p < 2.22e-16)	0.2225 (p < 2.22e-16)	0.3105 (p < 2.22e-16)	1.3581 (p = 1.3e-09)	1.0975 (p = 0.046)
BID, 50/25mg	0.2501 (p < 2.22e-16)	0.2069 (p < 2.22e-16)	0.2599 (p < 2.22e-16)	1.1515 (p = 0.0047)	1.0751 (p = 0.12)
