# Supplementary material for: Mathematical Modeling Identifies Optimum Palbociclib-fulvestrant Dose Administration Schedules for the Treatment of Patients with Estrogen Receptor–positive Breast Cancer
Source: Cancer Res Commun. 2023 Nov 16;3(11):2331–44. doi: 10.1158/2767-9764.CRC-23-0257 (PMC10652811; doi:10.1158/2767-9764.CRC-23-0257)
Supplement: Supplementary Appendix S1 — shows multistage model [file crc-23-0257-s20.pdf]

## Appendix. S1 Multistage model.

*Set up of multistage model* - To model *in vitro* cell dynamics, we considered that every cell follows a multistage Markov process of cell cycle progression [1] following a chain of transitions

$$s_1 \xrightarrow{\lambda_1} s_2 \xrightarrow{\lambda_2} \cdots \xrightarrow{\lambda_{n-1}} s_n \xrightarrow{\lambda_n} 2s_1, \tag{1}$$

where $s_k,\ k = 1, \cdots n$, represents the state of a single cell. The parameter $\lambda_k$ represents the transition rate from stage $k$ to the next stage, $k + 1$. Beginning from stage 1, a cell goes through all transitions with rates $\lambda_1, \cdots, \lambda_n$ before returning to stage 1 to complete the cell cycle. The factor 2 in front of $\lambda_n$ is to describe cell division after the completion of one cycle. By *the law of large numbers* (LLN), the growth dynamics of a large number cells that follow the chain of transition (4) can be described by a system of *ordinary differential equations* (ODEs)

$$\frac{\mathrm{d}x_k(t)}{\mathrm{d}t} = \begin{cases} 2\lambda_n x_n(t) - \lambda_1 x_1(t), & \text{for } k = 1, \\ \lambda_{k-1}x_{k-1}(t) - \lambda_k x_k(t), & \text{for } k = 2, \cdots, n, \end{cases} \tag{2}$$

where $\mathbf{x}(t) = (x_1(t), x_2(t), \cdots, x_n(t))$ represents the number of cells in stages $k = 1, 2, \cdots, n$ at time $t$. By summing over the number of cells in all stages given by the solutions of the system of ODEs (2), we have $x_T(t) = \sum_{k=1}^{n} x_k(t)$ to describe the dynamics of total cell growth. To incorporate a more detailed description of cell growth, we divided the $n$ stages of the cell cycle into $n_\alpha, n_\beta$, and $n_\gamma$ phases such that

$$x_\alpha(t) = \sum_{k=1}^{n_\alpha} x_k(t), \qquad x_\beta(t) = \sum_{k=n_\alpha+1}^{n_\alpha+n_\beta} x_k(t), \qquad x_\gamma(t) = \sum_{k=n_\alpha+n_\beta+1}^{n_\alpha+n_\beta+n_\gamma} x_k(t), \tag{3}$$

where $x_\alpha(t)$ represents the number of cells in phase G0/G1 of the cell cycle, $x_\beta(t)$ represents the number of cells in S phase, and $x_\gamma(t)$ represents the number of cells in G2/M at time $t$. In the most general case of the model, $n_\alpha, n_\beta, n_\gamma$, could be all different to represent various numbers of "subphases" of each cell-cycle phase. In order to avoid overfitting, we made two assumptions: (1) the three phases share the same number of subphases $m := n_\alpha = n_\beta = n_\gamma$ and (2) all subphases within each phase share the same transition rate $\lambda_\alpha$, $\lambda_\beta$, and $\lambda_\gamma$ for G0/G1, S, and G2/M phases, respectively.

*Examples of multistage Markov processes* - Multistage Markov processes can be viewed as a generalization of pure birth Markov processes, which simply assume $n = 1$ for chain

$$s_1 \xrightarrow{\lambda_1} s_2 \xrightarrow{\lambda_2} \cdots \xrightarrow{\lambda_{n-1}} s_n \xrightarrow{\lambda_n} 2s_1, \tag{4}$$

where $s_k,\ k = 1, \cdots n$, represents the state of a single cell. Parameter $\lambda_k$ represents the transition rate from the stage $k$ to the next stage $k + 1$. To begin with stage 1, a cell goes through all of the transitions with rates $\lambda_1, \cdots, \lambda_n$ then back to stage 1 to complete a cell cycle. Another special case of multistage Markov processes by taking $n = 3$ is equivalent to the classical cell-cycle model, in which $s_1$ is the cell state in G0/G1 phase, $s_2$ is the cell state in S phase, and $s_3$ is the cell state in G2/M phases. One key property of Markov processes is that each step has to be memoryless. However, a cell cycle is a complex process of biochemical reactions with a sequence of underlying rate-limiting steps, so it is not necessarily to presume one step (pure birth process) or three steps (classical cell-cycle model). Beyond those two cases, we provided more general assumptions in the next section for how to determine the number of stages $n$ and the transition rates $\lambda_k$ in modeling of cell cycle.

*Justifications of the model assumptions* - While our goal was to generalize our model to better describe our data, we also needed to avoid overfitting from a too complicated model. Therefore, we made two assumptions to reduce the dimension of space of

parameters: (1) Assume $m := n_\alpha = n_\beta = n_\gamma$, i.e. the numbers of subphases of three cell-cycle phases, G0/G1, S, G2/M, are equal and denoted by $m$. Inferring $m$ from our data can tell us the most possible number of underlying subphases of each phase for the cell line used in the present work (see next section). (2) Assume the expected duration of all subphases within their corresponding cell-cycle phase are equal. However, the three cell-cycle phases themselves could have distinct expected durations $\mu_\alpha, \mu_\beta$, and $\mu_\gamma$. Therefore, the corresponding expected durations become $\mu_\alpha/m, \mu_\beta/m$, and $\mu_\gamma/m$ for the subphases in G0/G1, S, and G2/M phases, respectively. By the inverse relation of the expected duration and the transition rate, we then derived three parameters

$$\lambda_\alpha = \frac{m}{\mu_\alpha}, \quad \lambda_\beta = \frac{m}{\mu_\beta}, \quad \lambda_\gamma = \frac{m}{\mu_\gamma}, \tag{5}$$

in which $\lambda_\alpha$ is the transition rate shared by all subphases in G0/G1, $\lambda_\beta$ is the transition rate shared by all subphases in S, and $\lambda_\gamma$ is the transition rate shared by all subphases in G2/M. From Eq. (5), we only required four parameters $m$, $\lambda_\alpha$, $\lambda_\beta$, $\lambda_\gamma$ for the multistage modeling of cell cycle progression. In comparison with the classical cell-cycle model, our simplified multistage model only added one additional degree of freedom in the parameter space, which is $m$. Similar assumptions were suggested in [2, 3], which showed this type of simplified multistage model gives a significant improvement in characterizing certain nonlinear behaviors (oscillations) of data without using a large dimension of free parameter space.

## References


1. Yates CA, Ford MJ, Mort RL. A multi-stage representation of cell proliferation as a Markov process. Bulletin of mathematical biology. 2017;79(12):2905–2928.

2. Vittadello ST, McCue SW, Gunasingh G, Haass NK, Simpson MJ. Mathematical models incorporating a multi-stage cell cycle replicate normally-hidden inherent synchronization in cell proliferation. Journal of the Royal Society Interface. 2019;16(157):20190382.

3. Gavagnin E, Vittadello ST, Gunasingh G, Haass NK, Simpson MJ, Rogers T, et al. Synchronized oscillations in growing cell populations are explained by demographic noise. Biophysical Journal. 2021;120(8):1314–1322.
