# Supplementary material for: Mathematical Modeling Identifies Optimum Palbociclib-fulvestrant Dose Administration Schedules for the Treatment of Patients with Estrogen Receptor–positive Breast Cancer
Source: Cancer Res Commun. 2023 Nov 16;3(11):2331–44. doi: 10.1158/2767-9764.CRC-23-0257 (PMC10652811; doi:10.1158/2767-9764.CRC-23-0257)
Supplement: Supplementary Appendix S2 — shows Bayesian estimation of model parameters [file crc-23-0257-s21.pdf]

Appendix. S2 Bayesian estimation of model parameters.

For the priors of the initial conditions $x_k(0)$ in the differential equations

$$\frac{\mathrm{d}x_k(t)}{\mathrm{d}t} = \begin{cases} 2\lambda_\gamma x_{3m}(t) - \lambda_\alpha x_1(t), & \text{for } k = 1, \\ \lambda_\alpha x_{k-1}(t) - \lambda_\alpha x_k(t), & \text{for } k = 2, \cdots, m-1 \\ \lambda_\alpha x_{m-1}(t) - \lambda_\alpha^*(d_F, d_P) x_m(t), & \text{for } k = m, \\ \lambda_\alpha^*(d_F, d_P) x_m(t) - \lambda_\beta x_{m+1}(t), & \text{for } k = m+1, \\ \lambda_\beta x_{k-1}(t) - \lambda_\beta x_k(t), & \text{for } k = m+2, \cdots, 2m, \\ \lambda_\beta x_{2m}(t) - \lambda_\gamma x_{2m+1}(t), & \text{for } k = 2m+1, \\ \lambda_\gamma x_{k-1}(t) - \lambda_\gamma x_k(t), & \text{for } k = 2m+2, \cdots, 3m, \end{cases} \tag{1}$$

we presumed that they are evenly distributed within each phase, i.e.

$$x_k(0) = \begin{cases} \frac{x_\alpha(0)}{m} & for\ k = 1, \cdots, m, \\ \frac{x_\beta(0)}{m} & for\ k = m+1, \cdots, 2m, \\ \frac{x_\gamma(0)}{m} & for\ k = 2m+1, \cdots, 3m. \end{cases} \tag{2}$$

Then we used relatively informative priors for $x_\alpha(0), x_\beta(0), x_\gamma(0)$ because we know what the initial cell population on day 0 from our data. Therefore, the most rational priors are centered around the mean of the observed cell cycle data on day 0, with standard deviations one order of magnitude smaller than the mean as

$$x_\alpha(0) \sim N(3000, 300) \tag{3}$$

$$x_\beta(0) \sim N(300, 300) \tag{4}$$

$$x_\gamma(0) \sim N(500, 300). \tag{5}$$

Based on the above priors (2)-(5), posteriors of $x_k(0)$, for $k = 1, \cdots 3m$, were estimated separately for each experiment, allowing for differences in plating.

Three chains were run so that we could evaluate chain convergence; each chain was run for 2000 iterations, with a warm-up period of 1000 to achieve a total of 1000 posterior samples to use for downstream simulations. The control parameter settings **adapt_delta** = 0.95, stepsize = 0.01 were used to avoid divergent transitions. The default values were used for all other control parameters. Posterior samples $\widehat{x_k}(t; d_F, d_P)$ were obtained by solving the system of differential equations listed in (1) using the posterior parameter values sampled by the MCMC. Posterior samples for the cell cycle phase count were obtained by

$$\widehat{x_\alpha}(t; d_F, d_P) = \sum_{k=1}^{m} \widehat{x_k}(t; d_F, d_P), \tag{6}$$

$$\widehat{x_\beta}(t; d_F, d_P) = \sum_{k=m+1}^{2m} \widehat{x_k}(t; d_F, d_P), \tag{7}$$

$$\widehat{x_\gamma}(t; d_F, d_P) = \sum_{k=2m+1}^{3m} \widehat{x_k}(t; d_F, d_P), \tag{8}$$

and posterior samples for the total cell count were obtained by

$$\widehat{x_T}(t; d_F, d_P) = \sum_{k=1}^{3m} \widehat{x_k}(t; d_F, d_P). \tag{9}$$

To account for noisy measurements, we assumed that the data observations $x_j^{(obs)}$, for $j \in \{\alpha, \beta, \gamma, T\}$, were sampled from normal distributions centered around the posterior estimates with standard deviation σ_j, i.e.

$$x_\alpha^{(obs)}(t; d_F, d_P) \sim \mathcal{N}(\widehat{x_\alpha}(t; d_F, d_P), \sigma_\alpha), \tag{10}$$

$$x_\beta^{(obs)}(t; d_F, d_P) \sim \mathcal{N}(\widehat{x_\beta}(t; d_F, d_P), \sigma_\beta), \tag{11}$$

$$x_\gamma^{(obs)}(t; d_F, d_P) \sim \mathcal{N}(\widehat{x_\gamma}(t; d_F, d_P), \sigma_\gamma), \tag{12}$$

$$x_T^{(obs)}(t; d_F, d_P) \sim \mathcal{N}(\widehat{x_T}(t; d_F, d_P), \sigma_T). \tag{13}$$

This assumption of normality is justified by the Central Limit Theorem. The prior distributions, initial parameter distributions, and lower and upper bounds are listed in **??**. The prior distributions for model parameters were chosen to be weakly informative. The initial parameter distributions were chosen so that the log likelihood could be calculated using the initial parameter values. Lastly, with the posterior parameters values $\widehat{\sigma_j}$ sampled by the MCMC, posterior predictive samples $x_j^{(pred)}$ were generated according to:

$$x_\alpha^{(pred)}(t; d_F, d_P) \sim \mathcal{N}(\widehat{x_\alpha}(t; d_F, d_P), \widehat{\sigma_\alpha}), \tag{14}$$

$$x_\beta^{(pred)}(t; d_F, d_P) \sim \mathcal{N}(\widehat{x_\beta}(t; d_F, d_P), \widehat{\sigma_\beta}), \tag{15}$$

$$x_\gamma^{(pred)}(t; d_F, d_P) \sim \mathcal{N}(\widehat{x_\gamma}(t; d_F, d_P), \widehat{\sigma_\gamma}), \tag{16}$$

$$x_T^{(pred)}(t; d_F, d_P) \sim \mathcal{N}(\widehat{x_T}(t; d_F, d_P), \widehat{\sigma_T}). \tag{17}$$

The R packages bayesplot [1, 2] and rstanarm [3] were used to visualize the posterior distribution and evaluate the model inference. Different rates were estimated for the -DOX (fulvestrant sensitive) and +DOX (fulvestrant resistant) cells. Also, we compared models with different m using the leave-one-out information criterion (LOOIC) [4] in order to infer the most probable number of subphases inside each phase. Model convergence was evaluated using the Potential Scale Reduction Factor ($\hat{R}$), which compares the estimated between-chains and within-chain variances for each parameters [5, 6]. The chains are considered converged if $\hat{R} < 1.1$ for all model parameters [6]. Effective sample size was used to measure the amount autocorrelation increased estimation uncertainty [7, 8], where an effective sample size of 0.1 of the total number of iterations is considered to be acceptable. The autocorrelation ρ_t measures the correlation between two chains offset by lag $t \geq 0$ positions, and is defined by

$$\rho_t = \frac{1}{\sigma^2} \int_\Theta \theta^{(n)} \theta^{(n+1)} p(\theta) \mathrm{d}\theta, \tag{18}$$

where $p(\theta)$ is the joint probability function of the chain and σ^2 is the chain variance. The effective sample size of N samples generated by a process with autocorrelation ρ_t is

$$N_{eff} = \frac{N}{\sum_{t=-\infty}^{\infty} \rho_t}. \tag{19}$$

References

1. Gabry J, Mahr T. bayesplot: Plotting for bayesian models;.
2. Gabry J, Simpson D, Vehtari A, Betancourt M, Gelman A. Visualization in Bayesian workflow. Journal of the Royal Statistical Society: Series A (Statistics in Society). 2019;182(2):389–402.
3. Goodrich B, Gabry J, Ali I, Brilleman S. rstanarm Bayesian applied regression modeling via Stan.;.
4. Vehtari A, Gelman A, Gabry J. Practical Bayesian model evaluation using leave-one-out cross-validation and WAIC. Statistics and computing. 2017;27(5):1413–1432.
5. Gelman A, Rubin DB. Inference from iterative simulation using multiple sequences. Statistical science. 1992;7(4):457–472.
6. Brooks SP, Gelman A. General methods for monitoring convergence of iterative simulations. Journal of computational and graphical statistics. 1998;7(4):434–455.
7. Geyer CJ. Introduction to markov chain monte carlo. Handbook of markov chain monte carlo. 2011;20116022:45.
8. Gelman A, Carlin JB, Stern HS, Rubin DB. Bayesian data analysis. Chapman and Hall/CRC; 1995.
