# Supplementary material for: Mathematical Modeling Identifies Optimum Palbociclib-fulvestrant Dose Administration Schedules for the Treatment of Patients with Estrogen Receptor–positive Breast Cancer
Source: Cancer Res Commun. 2023 Nov 16;3(11):2331–44. doi: 10.1158/2767-9764.CRC-23-0257 (PMC10652811; doi:10.1158/2767-9764.CRC-23-0257)
Supplement: Supplementary Appendix S3 — shows multistage model versus logistic model [file crc-23-0257-s22.pdf]

Appendix. S3 Multistage model versus logistic model.

To include the effect of carry capacity, we can use the a three-compartment system of ordinary differential equations representing the G0/G1, S, and G2/M phases with logistic growth in each phase, given by

$$\begin{aligned} T &= x_\alpha(t) + x_\beta(t) + x_\gamma(t), \\ \frac{\mathrm{d}x_\alpha(t)}{\mathrm{d}t} &= 2\lambda_\gamma x_\gamma \left(1 - \frac{T}{K}\right) - \lambda^*_\alpha(d_F, d_p) x_\alpha \left(1 - \frac{T}{K}\right), \\ \frac{\mathrm{d}x_\beta(t)}{\mathrm{d}t} &= \lambda^*_\alpha(d_F, d_p) x_\alpha \left(1 - \frac{T}{K}\right) - \lambda_\beta x_\beta \left(1 - \frac{T}{K}\right), \\ \frac{\mathrm{d}x_\gamma(t)}{\mathrm{d}t} &= \lambda_\beta x_\beta \left(1 - \frac{T}{K}\right) - \lambda_\gamma x_\gamma \left(1 - \frac{T}{K}\right), \end{aligned} \tag{1}$$

where x_α, x_β, and x_γ represent the number of cells in G1, S, and G2/M phases of the cell cycle, respectively; λ_γ denotes the G2/M to G1 transition rate (day^{-1}); $\lambda^*_\alpha(d_F, d_p)$ following

$$\lambda^*_\alpha(d_F, d_P) = \lambda^{(max)}_\alpha + (\lambda_\alpha - \lambda^{(max)}_\alpha) r_F(d_F) r_P(d_P), \tag{2}$$

denotes the dose dependent G1 to S transition rate (day^{-1}) where d_F and d_P are the concentrations of fulvestrant and palbociclib, respectively; λ_β represents the S to G2/M transition rate (day^{-1}); and K represents the carrying capacity (number of cells).

Then we used the model comparison (LOOIC) to compare the logistic growth model given by (1) and the multistage model given by

$$\begin{aligned} \frac{\mathrm{d}x_\alpha(t)}{\mathrm{d}t} &= 2\lambda_\gamma x_{3m}(t) - \lambda^*_\alpha(d_F, d_P) x_m(t), \\ \frac{\mathrm{d}x_\beta(t)}{\mathrm{d}t} &= \lambda^*_\alpha(d_F, d_P) x_m(t) - \lambda_\beta x_{2m}(t), \\ \frac{\mathrm{d}x_\gamma(t)}{\mathrm{d}t} &= \lambda_\beta x_{2m}(t) - \lambda_\gamma x_{3m}(t). \end{aligned} \tag{3}$$

Two models have the same number of parameters: the only difference is that the logistic growth model has K for the carry capacity but the multistage model has m for the number of subphases. All of the rest of parameters are the same. So we have a similar model complexity (the same dimension of parameter space), which makes the model comparison reliable [1]. Our result of model comparison suggested that the multistage model is better than the logistic growth model in terms of predictive analytics, for both -DOX and +DOX cells. See Table 1.

Table 1. Model comparison by LOOIC.

	**Logistic model**	**Multistage model**
-DOX	-2209.5	-2239
+DOX	-1776	-2057.3

The values are given by LOOIC $= -2\text{epld}_{\text{loo}}$, where epld_{loo} represents the Bayesian LOO estimate of expected log predictive density. Since there is a factor -2 in the formula (historical reasons), a smaller, more negative number indicates of LOOIC better predictions [2].

References

1. McElreath R. Statistical rethinking: A Bayesian course with examples in R and Stan. Chapman and Hall/CRC; 2020.

2. Vehtari A, Gelman A, Gabry J. Practical Bayesian model evaluation using leave-one-out cross-validation and WAIC. Statistics and computing. 2017;27(5):1413–1432.
